# Exploring the association between serotonin transporter promoter region methylation levels and depressive symptoms: a systematic review and multi-level meta-analysis

**DOI:** 10.1038/s41398-025-03356-w

**Published:** 2025-05-03

**Authors:** F. Javelle, G. Dao, M. Ringleb, W. Pulverer, W. Bloch

**Affiliations:** 1 NeuroPsychoImmunology research unit, Department for Molecular and Cellular Sports Medicine, Institute for Cardiovascular Research and Sports Medicine, German Sport University Cologne, Cologne, Germany; 2 University of Cologne, Cologne, Germany; 3 Department of Movement Science, University of Münster, Münster, Germany; 4 Department of Sports Medicine and Health Promotion, Friedrich-Schiller-University Jena, Jena, Germany; 5 Austrian Institute of Technology, Vienna, Austria; 6 Department for Molecular and Cellular Sports Medicine, Institute for Cardiovascular Research and Sports Medicine, German Sport University Cologne, Cologne, Germany

**Keywords:** Clinical genetics, Human behaviour

## Abstract

Depressive disorders result from complex interactions among genetic, epigenetic, and environmental factors. DNA methylation, a key epigenetic mechanism, is crucial in understanding depressive symptoms development. The serotonin transporter gene (*5-HTT*) and its polymorphisms, like *5-HTTLPR*, have been extensively studied in relation to depression, yet conflicting findings regarding the association between 5-HTT promoter methylation and depressive symptoms persist, largely due to methodological differences. Thus, this systematic review and meta-analysis aims to assess (1) *5-HTT* promoter methylation levels between depressed and non-depressed conditions and (2) the association between *5-HTT* methylation and depressive symptoms severity. We searched PubMed, Google Scholar, and Web of Science from inception to January 15th, 2025 (PROSPERO: CRD42023355414) and performed two independent multi-level meta-analyses to answer our aims. Twenty-four trials were included in the systematic review. All reported effects carried potential for bias. The meta-analysis for depression occurrence (12 studies - 2028 subjects – 127 effects) indicated no significant effect (Hedges’g = 0.06) with moderate within- and low between-study heterogeneity. The depression severity analysis (14 studies - 2296 subjects - 116 effects) revealed a null effect size (Fisher’s Z = 0.05), with no within- and moderate between-study heterogeneity. Asymmetry was detected for both meta-analyses. Moderator analyses demonstrated no significant effects of depression severity, methylation techniques, single-CpG sites, cell types assessed, age, and female percentage. This comprehensive review provides insights into the intricate interplay between *5-HTT* promoter methylation and depressive symptoms. Furthermore, it offers well-considered recommendations for future research endeavors and delineates guidelines for reporting methylation research.

## Introduction

Depressive disorders are complex disabling mental disorders with an estimated prevalence of 3.8% of the world population, rising to 5.1% in adults in 2021 [1, 2]. Despite extensive research, the precise etiology of depression remains unclear and finds ties with multiple endocrine, immune, and metabolic mechanisms at functional, genomic, and epigenomic levels impacting brain functions and structure [3–6]. Yet, recent research underlined the crucial importance of epigenetic modifications, such as DNA methylation, in the development of depressive symptoms [7–9].

The serotonin transporter (*5-HTT* or *SLC6A4*) gene has been widely studied in relation to depression, as it encodes the protein responsible for transporting serotonin, a neurotransmitter involved in multiple functions but widely accepted for its role in mood regulation [10, 11]. For example, the promoter region of the *5-HTT* gene contains a polymorphism known as the Serotonin Transporter-Linked Polymorphic Region (*5-HTTLPR*), which has been associated with both depression susceptibility and treatment response [12–14]. The different variants of this polymorphism arise from combinations of two alleles, which can be either short or long, with, if the allele is long, a single nucleotide polymorphism (rs25531) further dividing it into two different forms. These variants have been shown to result in distinct *5-HTT* transcriptional activity levels and, therefore, distinct subsequent *5-HTT* protein levels [11, 15, 16]. Recently, studies have shown that the transcriptional activity levels of *5-HTT* are strongly affected by its methylation levels [17, 18]. Methylation is the addition of a methyl group (CH3) on the cytosine of cytosine-guanine dinucleotides (CpG) on deoxyribonucleic acid (DNA) sequences [19, 20]. Gene promoters have cytosine-enriched regions called CpG islands that are mostly affected by methylation [19, 20]. In most cases, high methylation levels in the promoter region relate to lower gene transcriptional activity, thus lowering the quantity of the subsequently synthesized protein potentially important for human health and behavioral control [20, 21]. Therefore, multiple studies have investigated the relationship between methylation levels in the *5-HTT* promoter region and depressive symptoms (e.g., [14, 22, 23]).

While some studies have suggested that hypermethylation of the *5-HTT* promoter region is associated with an increased risk of depression [22, 24, 25], others reported conflicting findings [26–28]. Furthermore, studies examining the association between *5-HTT* promoter region methylation levels and depression utilized different techniques for measuring methylation levels, such as bisulfite sequencing (e.g., [27]), bisulfite pyrosequencing (e.g., [29]), Matrix-Assisted Laser Desorption/Ionization-based time of flight (MALDI-TOF) methylation (e.g., [28]) or microarray (e.g., [30]). Additionally, different regions of the *5-HTT* promoter have been analyzed, ranging from the core promoter region to more distal regions containing more or less CpG sites. These methodological differences may contribute to heterogeneity across studies and highlight the need for a systematic review and meta-analysis to evaluate the consistency and strength of the association between *5-HTT* promoter region methylation levels and depressive symptoms.

Moreover, despite the importance of gene expression in depression pathogenesis, few studies have assessed the relationship between *5-HTT* promoter region methylation and gene expression [9, 22, 31, 32]. Even rarer are the studies that evaluate cell-specific methylation instead of whole blood methylation levels. These points underline the interest in this relatively new epigenetic field but emphasize the poor understanding of the underlying mechanisms linking epigenetics and behavior. Thus, a comprehensive evaluation of the relationship between *5-HTT* promoter region methylation levels, gene expression, and depressive symptoms is necessary to elucidate the underlying epigenetic mechanisms of depression further.

This systematic review and multi-level meta-analysis aims to examine the association between *5-HTT* promoter region methylation levels and depressive symptoms in humans. We conducted two meta-analyses assessing (1) *5-HTT* promoter methylation levels between depressed and non-depressed conditions and (2) the association between *5-HTT* methylation and the severity of depressive symptoms. We aim to provide an overview of the current evidence on this topic and explore potential heterogeneity sources across studies. We hypothesized that depressed individuals would have *5-HTT* promoter region more methylated than non-depressed individuals, that there would be a positive association between *5-HTT* promoter region and depression severity, and that the type of samples used would particularly modulate those effects. Our findings may contribute to a better understanding of the epigenetic mechanisms underlying depression and have implications for developing novel therapeutic strategies.

## Materials and methods

The literature search and writing process were conducted in accordance with the Preferred Reporting Items for Systematic Reviews and Meta-Analyses (PRISMA) guidelines [33, 34]. The PRISMA checklist is provided in Supplementary Material A. The protocol was pre-registered on PROSPERO (CRD42023355414) before the analysis (last edited version: 29/08/2023). Protocol, script, R_markdown_ file (Supplementary B), and data are freely available on Open Science Framework (https://osf.io/guatx/?view_only=de7aafa4cad848ca97ab052086b7bf10).

### Literature source and study selection

**Fig. 1:**
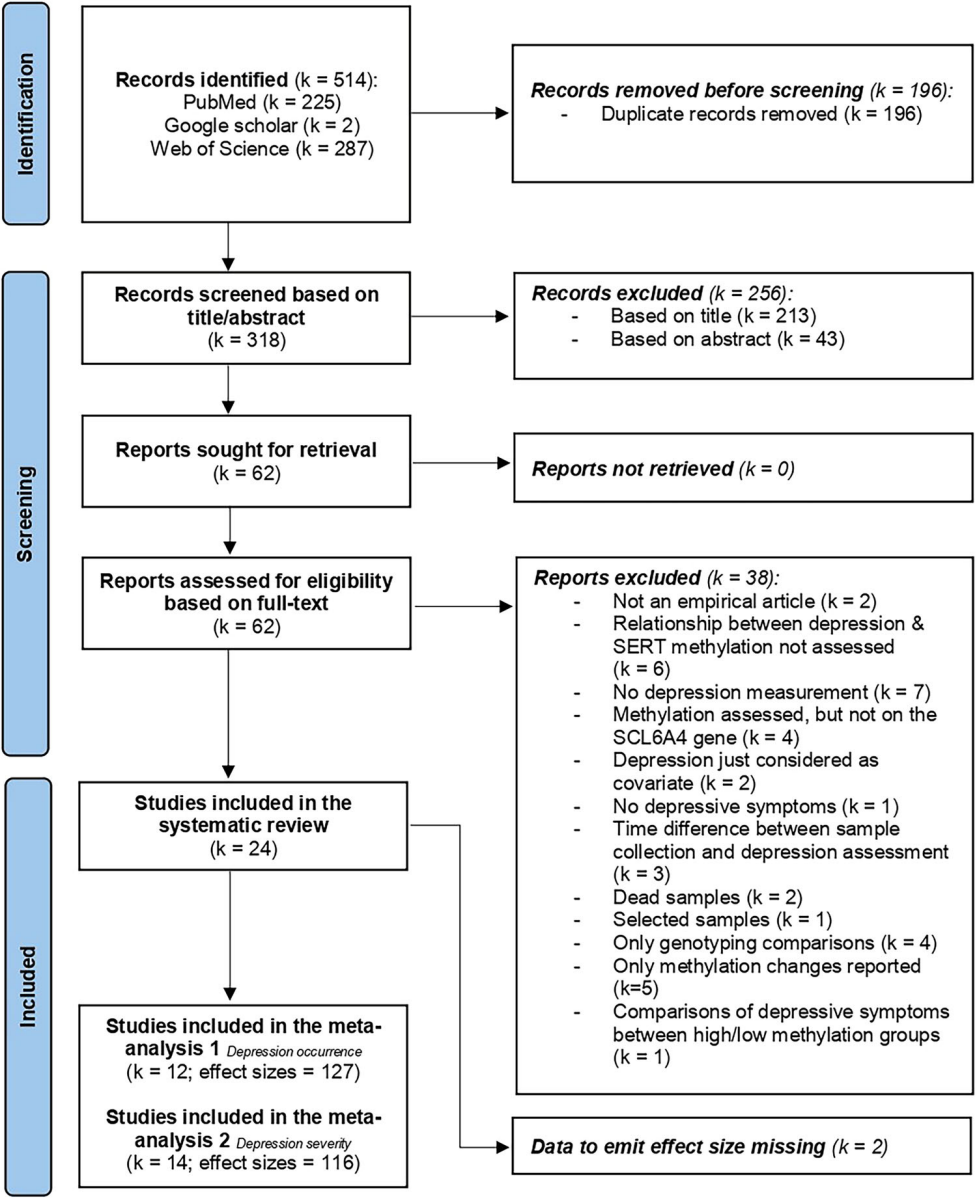
Flow diagram of the study review process. The literature search was conducted using PubMed, Google Scholar, and Web of Science, covering all records up to January 15, 2025. K indicates the number of studies. SERT and SLC6A4 refer to the serotonin transporter gene.

The literature search was conducted using PubMed, Google Scholar, and Web of Science from inception to January 15th, 2025. The search strategy was created from three Medical Subject Headings (MeSH - Methylation, *SLC6A4*, and Depression) and text words combined through Boolean operators (“AND”, “OR” - Table 1). The search string was adjusted according to the formal requirements of each database. Only peer-reviewed articles published in English were included in the review. No limitations were applied for the publication year. Additionally, cross-referencing of the selected articles was performed. After removing the duplicates, studies were screened for eligibility by two independent researchers (G.D and M.R) in a three-step process based on title, abstract, and full text using the reference manager Mendeley (Fig. 1). Screening techniques and eligibility criteria were first discussed. Then, 20 random articles were first screened independently, and results were compared between reviewers to avoid any discrepancy in the selection process. Any doubts or differences were clarified with the help of a senior reviewer (F.J).

**Table 1:** Example of a search string for the database of PubMed/MEDLINE.

**Database:** PubMed/MEDLINE
Methyl* [Title/Abstract] AND (5-HTT [Title/Abstract] OR 5-HTTLPR [Title/Abstract] OR serotonin- linked polymorphic region [Title/Abstract] OR SLC6A4 [Title/Abstract] OR serotonin transporter [Title/Abstract]) AND (Depress* [Title/Abstract] OR MDD [Title/Abstract])
**MeSH terms:** Methylation, SLC6A4, Depression

### PICOS criteria

#### Participants

For both analyses, the sample of interest in this meta-analysis was adults/children (no sex or age restriction) with depressive symptoms. Depressive symptoms could have been recorded in the frame of a depressive disorder or as comorbid symptoms from another condition. Animal studies and after-death methylation analyses were excluded from this report.

#### Intervention

Interventions or situations considered were analyses of methylation levels and depression severity at rest (on the same day or one day apart). Acute stimulations of the patients (psychologically or physically) just before blood gathering or depressive diagnosis were excluded from this report.

#### Comparison

For the assessment of 5-HTT promoter methylation levels between depressed and non-depressed conditions, controls were either healthy individuals, individuals with the same condition but without depressive symptoms, or participants in longitudinal studies with pre- or post-depression assessments. For the evaluation of the association between 5-HTT methylation and depressive symptom intensity, cohorts with varying levels of depression severity were included.

#### Outcomes

First, to understand if individuals suffering from depressive symptoms have higher *5-HTT* gene methylation levels than healthy controls, methylation levels of *5-HTT* promoter between individuals with and without depressive symptoms were compared. Second, to evaluate if depressive symptoms’ severity relates to methylation levels in the *5-HTT* gene, correlation, partial correlation, regression, or multiple regression between depressive symptoms intensity and methylation levels of *5-HTT* were used.

#### Study design

Longitudinal and cross-sectional studies were considered in this report. Single-case studies were excluded from this report.

### Data extraction

The selection process was performed using the reference manager software Mendeley. Studies were successively assessed based on title, abstract, and full text. For each study, (1) the number of participants in each group, (2) methylation levels (global and per CpG site) with mean and standard deviation (or standard error of the mean or 95% confidence interval), (3) the study design, (4) the variables controlled for, (5) the type of depression, (6) primary/comorbid disorders, (7) depression severity, (8) depression assessment tool, (9) biological samples used for the methylation analysis, (10) the methylation sites, (11) the number of CpG sites assessed, (12) the methylation technique used, (13) if the expression level was assessed and the technique used, (14) age, (15) sex, and (16) ethnicity were extracted.

If the authors did not provide exact values, the WebPlotDigitizer digitization program (https://automeris.io/WebPlotDigitizer/) was used to extract plotted data. The corresponding author of the article was also contacted to obtain the missing data.

### Quality assessment of the included studies

The Joanna Briggs Institute (JBI) has developed a suite of critical appraisal tools for systematic reviews, including the Checklist for Analytical Cross-Sectional Studies [35]. This tool consists of eight items covering important aspects, including (1) exclusion/inclusion criteria, (2) participants and study setting description, (3) measurement of exposure, (4) condition and (7) outcome, (5) existence of confounding factors and (6) strategy to deal with it and finally, (8) appropriate statistical measures. The rating focused on the outcome of interest for our meta-analyses, and was therefore conducted twice if studies were included in both meta-analyses. Since we included papers where the extracted outcomes were not always the primary focus of the study, it is important to note that the grading was based on those specific outcomes rather than the study’s main objective.

For Item 1, the outcome was considered as ‘not applicable’ (n/a) if the sample had been drawn from a larger study. To meet the criteria of Item 2, essential information included age (mean ± SD or median with range), gender distribution (frequency), ethnicity, medication status (at least indicating medicated or not), depression severity, and the presence of comorbidities. Concerning Item 3, the emphasis was on DNA extraction. Fulfilling this item necessitated providing minimal descriptions of the biological samples utilized, extraction techniques employed, and the details of quality control measures. Meeting the requirements of Item 4 entailed using validated questionnaires or clinician interviews for diagnosing or assessing depression. For Items 5 and 6, which focused on group differences, ‘not applicable’ ratings were assigned to studies included in the meta-analysis evaluating depression severity and *5-HTT* methylation. To satisfy the criteria of Item 7, the methylation techniques employed needed to have the capability of single-site assessment, the genomic location had to be clearly reported (including the used assembly) and valid assessments of depression needed to be incorporated. An ‘unclear’ rating was given if the reported methylation technique offered the single-site option, but only average scores were reported. Finally, for Item 8, if the extracted outcomes were not the primary focus of the study and no statistical tests were conducted, the emphasis shifted towards assessing the distribution of the data.

At the beginning of the grading process, members of the reviewing team discussed the scale and agreed on how to grade each item. Then, two researchers (G.D, F.J) independently rated studies and obtained an inter-rater correlation coefficient of 90.4%. Disagreements were then solved by discussion.

### Effect sizes

To assess *5-HTT* promoter methylation levels between depressed and non-depressed conditions, all outcome measures were standardized using Hedges’g (SMD = MD/SD_pooled_) and its standard error according to the recommendations from Borenstein and colleagues ([36] equations 4.18 to 4.25 page 22 to 25). A positive g denotes a more important methylation of *SLC6A4* in the depressed group compared to healthy control in meta-analysis (1), and it denotes a positive association between depression severity and *SLC6A4* methylation in meta-analysis (2). Hedges’g (g) of 0.2, 0.5, and 0.8 are respectively considered small, moderate, and large [37, 38].

To assess the association between *5-HTT* methylation and the severity of depressive symptoms, the outcomes were standardized from rho to Fisher’s Z scale ([36], equation 6.2, page 42). The variance was first computed using the formula Vr = (1-rho^2^)^2^/n-1 ([36], equation 6.1, page 41) and then also transformed to a Fisher’s Z scale ([36], equations 6.3 and 6.4, page 42).

### Statistical analysis

Since trials tend to report multiple CpG methylation sites, statistical analyses were based on a three-level maximum likelihood random-effects model using the packages metaSEM and metafor for R (version 4.1.0). These models account for the dependency of effect sizes within studies by providing estimates of variance within (level 2 indexed below by I^2^_(2)_ = and τ^2^_(2)_) and between studies (level 3 indexed below by I^2^_(3)_ and τ^2^_(3)_) [39–41].

The heterogeneity within those levels was quantified using τ^2^ (variance of true effects, using Hedges’ estimator [38]) and further assessed using I^2^, which provides the percentage of the observed variance reflecting the variance of the true effects rather than sampling error [42]. The prediction interval was also computed to consider the potential association between *5-HTT* methylation and depressive symptoms when reported in an individual study setting, as this may be different from the average effect. Potential asymmetry was assessed by visually inspecting the funnel plots. Furthermore, when at least ten studies were available, the asymmetry was assessed using Egger’s test. If evidence for asymmetry was found (*p* < 0.1 on the Egger’s test), the Duval and Tweedie trim and fill method was used to quantify the magnitude of the small study effect [43]. To detect moderator effects, subgroup analyses were applied to the categorical variables of interest (i.e., depression severity, methylation techniques, cell types, ethnicity). Q-tests were performed to evaluate differences between subgroups [42]. Meta-regressions were used to analyze the moderating effect of the continuous variables [36] age and female percentage. The predictive value of this continuous moderator was evaluated by the goodness of fit (R²) and was significant at the *p* = 0.05 level.

## Results

### Study characteristics

Out of the 514 identified articles, 24 studies were included in the systematic review (Fig. 1). Two studies (413 participants) did not report and provide data, despite the authors being contacted for inclusion in the meta-analyses [9, 44]. One of these studies indicated a trend suggesting an association between increased overall methylation and a lifetime history of major depression [9], while the other found no significant associations between the eight tested CpG sites and depression severity [44]. Four out of the 24 studies were included in both meta-analyses. The meta-analysis assessing *5-HTT* promoter methylation in depressed individuals compared to healthy controls comprised twelve studies, with 127 effects and 2028 participants. The meta-analysis assessing the association between *5-HTT* promoter methylation and depression severity included 14 studies with 116 effects and 2296 participants.

The mean sample size and the average age of the patients were highly heterogeneous, with respective means and standard deviations of 169.3 (±176.3) individuals and 40.7 years old (±13.0 years old) (Table 2). The study by van der Knapp and colleagues stood out in both cases, involving 939 adolescents. Reflecting the sex distribution discrepancy, the average percentage of females per study was 59.3% (±21.1%). Two studies exclusively included males [32, 45] and another focused exclusively on females [30]. Seven studies did not report participants’ ethnicity [8, 24, 25, 31, 45–48]. Among those that did, reported ethnicities included European ancestry (k = 2) [27, 49], Caucasian (k = 5) [23, 28, 29, 32, 50], Dutch (k = 1) [51], Chinese Han (k = 2) [7, 26], Korean (k = 2) [44, 52], Japanese (k = 3) [14, 22], African-American (k = 2) [9, 30], Hispanic (k = 1) [9], and Rwandan (k = 1) [53].

**Table 2:** Studies description table.

First author, year	*N* tot _(ne, nc)_	Age	Sex _(female %)_	Ethnicity _(as reported in papers)_	Type of depression	Severity of depression	Primary disorder	Depression scale used	Comorbidity	Controls Used	Sample	Methylation technique	Single-CpG resolution option?	CpG Data Type Used for Effect Computation	Number of CpGs _(/74 CpG in the CpG island)_	Genomic location	GRCh38/hg38 conversion _UCSC Liftover - 30 23__ _ _ ___ _(for ref. 799 bp CpG island: 5 370 – 6 168)_	Study Effect Sizes	Expression tested?
**Meta-analysis 1 – Depression occurrence**_**Group comparisons**_ **(12 studies, 127 effects, 2028 participants)**																			
Bruzzone, 2024	387 _(90, 297)_	29.6	63.9	EUR	MDD	Moderate _all_	MDD	BDI – HAM-D_17_	Anx., chilhood trauma	Healthy	Blood _peri_ or WBC	Pyroseq. _PyroMark Q96_	Y	Single	4	GRCh38/hg38 _30,236,071;_ _30,236,083;_ _30,236,088;_ _30,236,090_	_6090; 6088;_ _6083; 6071_	4	N
Bakusic, 2020	138 _(80, 58)_	45.4	56.5	*n/a*	MDD	Moderate _average_	MDD	SCID	*n/a*	Healthy	Blood _peri_	Pyroseq. _PyroMark Q24_	Y	Single	23 CpG	GRCh38/hg38 _GenBank NG_011747_	_6101; 6090;_ _6088; 6083;_ _6071; 6036;_ _6002; 5998;_ _5984; 5975;_ _5973; 5968;_ _5956; 5952;_ _5939; 5910;_ _5906; 5904;_ _5897; 5892;_ _5888; 5886;_ _5871; 5867;_ _5852_	23	N
Schiele, 2019	60 _(32, 28)_	34.3	78.3	CA	Comord. MDD	*n/a*	PD	SCID	Ago., MDD, social Anx., phobias	PD without MDD	Blood _peri_	Pyroseq.	Y	Single	9	GRCh38/hg38 _30,236,071;_ _30,236,083;_ _30,236,088;_ _30,236,090;_ _30,236,101;_ _30,236,120;_ _30,236,125;_ _30,236,141;_ _30,236,156_	_6156; 6141;_ _6125; 6120;_ _6101; 6090;_ _6088; 6083;_ _6071_	9	N
Lam, 2018	$302_{{(95,\, {207})}^{\rm{b}}}$	73.2	62.5	CA	MDD	> Mild _all_ + Moderate _average_	MDD	MINI – CES-D	Dementia, Anx., ischemic diseases, cancer, thyroid disease, diabetes, asthma	Healthy elders	WBC	MALDI-based _Agena_	N	Averaged per unit	10 units/20 CpG	GRCh38/hg38 _**Reg.:**_ _30,235,734–_ _30,236,068_	_6036; 5984;_ _5975/73;_ _5956/52;_ _5939;_ _5909/891;_ _5886/84;_ _5870/60;_ _5795;_ _5768/65_	10	N
Schneider, 2018	298 _(122, 176)_	36.3	53	EUR	MDD	Moderate _average_	MDD	SCID	*n/a*	Healthy	Blood _peri_	BS - PCR _EZ-DNA methylation kit -ESME software_	Y	Single	8^d^	GRCh38/hg38 - AluJb	_6593; 6587;_ _6571; 6511;_ _6509; 6500;_ _6496;_	7	N
Shi, 2017	151 _(96, 55)_	31 _med._ _(21-47)_	60.3	CHA	MDD	Severe _all_	MDD	SCID – HAM-D_24_	*n/a*	Healthy	Blood _peri_	Pyroseq. _Pyro Q-CpG_	Y	Single	6	GRCh37/hg19 _28,562,957;_ _28,562,970;_ _28,562,974;_ _28,562,986;_ _28,562,991;_ _28,562,993_	_5975; 5973;_ _5968; 5956;_ _5952; 5939_	6	N
Iga, 2016	57 _(28, 29)_	43.6	71.9	JA	MDD	Moderate _average_	MDD	SCID	Diabetes, hypertension, gastritis	Healthy	WBC	Pyroseq. _PyroMark Q24_	Y	Single	9	GRCh37/ hg19 _GenBank NC_000017.10_	_6156; 6141;_ _6125; 6120;_ _6101; 6090;_ _6088; 6083;_ _6071_	9	Y
Won, 2016	84 _(35, 49)_	40.8	70.2	KA	MDD	Moderate _average_	MDD	SCID	Suicide, drug abuse	Healthy	Blood _peri_	Pyroseq. _Pyrosequencing Assay Design Software_	Y	Single	5	*n/a* – _−34 to −5 bp relative to the TSS_	_5732; 5728;_ _5714; 5705;_ _5703_	5	N
Booij, 2015	69 _(33, 36)_	37.7	63.8	*n/a*	MDD	Severe _all_	MDD	SCID	*n/a* _NO psychiatric comorb._	Healthy	Blood _peri_	Pyroseq. _PyroMark Q24_	Y	Average _all + specific sites_	11^d^	*n/a* _214 to 625 bp region upstream of TSS_	_6167–6036;_ _6167/56;_ _6090/88;_	3	Y
Okada, 2014	100 _(50, 50)_	40.3	46	JA	MDD	Moderate _all_	MDD	MINI	*n/a*	Healthy	Blood _peri_	MALDI-based _Agena_	N	Single	29 units / 47 CpG	GRCh37/hg19 _**Reg**.:_ _28,562,388–_ _28,563,186_	spe. CpG sites: *n/a* _**Reg:**_ _5 370 - 6 168_	29	N
Kim, 2013	286 _(80, 206)_	64.5	40.9	*n/a*	PSD	Moderate _average_	PSD	MINI	*n/a* _NO dementia, Parkinson, brain tumor, epilepsy, psychosis, substance dependence; severe physical illnesses limiting movement prior PSD_	Healthy	Blood _peri_	Pyroseq. _Pyro Q-CpG_	Y	Single	7	*n/a* _−479 to −350 bp relative to the TSS_	_6141; 6125;_ _6101; 6090;_ _6088; 6083;_ _6071_	7	N
Xu, 2013	96 _(36, 60)_	28.2	80.2	CHA	Comorb. depr.	Moderate _average_	SLE	SCID	Depr.	SLE without depr.	WBC	BS - PCR _Shanghai Sangon Biological Engineering Technology & Services Co. Ltd._	Y	Single	15 ^d^	*n/a* _GenBank: EF179203.1_	_7150; 7146;_ _7133; 7105;_ _7097; 7080;_ _7070; 7068;_ _7066; 7044;_ _7036; 7029;_ _7018; 7002;_ _6995_	15	N
**Systematic review only - Depression occurrence**_**Group comparisons**_ **(2 studies, 413 participants)**																			
Moon, 2022	221	64.0	78.2	KA	MDD	Moderate _all_	MDD	HAM-D_17_	*n/a* _NO cancer, unstable psychiatric features, history of substance abuse, neurologic illnesses, SCZ, BD, primary diagnoses of adjustment disorder, PTSD, psychotic sympt., pregnancy_	Only MDD	Blood _peri_	Pyroseq. _PyroMark_	Y	Single	9	GRCh37/ hg19 _28,563,090;_ _28,563,102;_ _28 563,107;_ _28,563,109;_ _28,563,120;_ _28,563,139;_ _28,563,144;_ _28,563,160;_ _28,563,175_	_6156; 6141;_ _6125; 6120;_ _6101; 6090;_ _6088; 6083;_ _6071_	-	Y
Philibert, 2008	192 _(25, 167)_	40.6	50	AFA, HA, others	MDD	*n/a*	MDD	SCID	Alcohol dependence	Healthy	lymphoblast cell lines	BS - PCR _MassARRAY_	N	-	53 units/ 71 CpG	NCBI36/hg18 _**Reg**.:_ _25,586,414 –_ _25,587,412_	_**Reg.:**_ _5270–6268_	-	Y
**Meta-analysis 2 – Depression severity**_**Correlations/Regressions**_ **(14 studies, 116 effects, 2296 participants)**																			
Rivera, 2024	91	24.0	50.5	RWD	Depr. Sympt. + MDD	Mild _average_	No diagnosis or MDD	PROMIS-29	Childhood adversity	MDD + Healthy	_capillary_ Blood _peri_	Microarray _Illumina Infinium MethylationEPIC microarray_	N _not in this case_	Average	*n/a*	*n/a*	*n/a*	1	N
Comtois-Cabana, 2023	156	24.1	0	*n/a*	Depr. Sympt. + MDD	Mild _average_	No diagnosis or MDD	BDI-II	Drug use	MDD + Healthy	Saliva	BS – PCR _EZ-DNA methylation Gold Kit - EpiTYPER MassARRAY_	Y	Average _per unit_	31 units/87 CpG	GRCH37/hg19 _**Reg. 1**:_ _28,563,054–_ _28,563,424;_ _**Reg. 2**:_ _28,562,783–_ _28,563,020;_ _**Reg. 3**:_ _28,562,388–_ _28,562,751_	_6406; 6235;_ _6167; 6141;_ _6036; 5896/91;_ _5886/84/70;_ _5866; 5851;_ _5845/43;_ _5837–5829;_ _5808, 5768/65;_ _5733/31;_ _5719–5699;_ _5688–5673;_ _5667/65; 5654;_ _5641; 5 578;_ _5554/49;_ _5518/11;_ _5503; 5489;_ _5481/74/71;_ _5456; 5447;_ _5417; 5394;_ _5383; 5374/70_	31	N
Vidovic, 2023	25	52.1	64.0	CA	MDD	Moderate to Severe _average_	MDD	BDI-II – IDS-C	Anx.	Only MDD	Blood _peri_	BS – PCR _EpiTect Fast Bisulfite - MiniSeq Mid Output Kit_	N	Average	2	GRCH37/hg19 _**Reg. 1**:_ _28,562,753–_ _28,563,050_ _**Reg. 2**:_ _28,563,277–_ _28,563,552_	_**Reg. 1:**_ _5735–6032_ _**Reg. 2:**_ _6259 –_ _6534_	2^c^	N
Timmers, 2022	117	46.6	65.8	*n/a*	Depr. Sympt. + MDD	Mild _average_	Dystonia	BDI	Depr.	Dystonia with/without Depr. Sympt.	Blood _peri_	Pyroseq. _PyroMark Q24_	Y	Single	20	*n/a* _−213 to −69 bp relative to the transcriptional start_	_5910; 5906;_ _5904; 5896;_ _5891; 5886;_ _5884; 5870;_ _5865; 5850;_ _5845; 5843;_ _5837; 5835;_ _5832; 5830;_ _5808; 5796;_ _5779; 5776_	20	N
Sanwald, 2021	146	38.7	65.1	*n/a*	MDD	Moderate to Severe _average_	MDD	BDI-II – MADRS	Alcohol abuse _(*n*=2)_, sexual dysfunction _(*n*=1)_	Only MDD	Blood _peri_	MALDI-based _Agena_	N	Average_2 factors_	29 units / 71 CpG	GRCh38/hg38 _**Reg. 1** :_ _30,235,345–_ _30,235,765_ _**Reg. 2:**_ _30,235,734–_ _30,236,068_	_**Reg. 1** :_ _5345–5765_ _**Reg. 2:**_ _5734–6068_	2^c^	N
Schiele, 2021	236	34.3	78.3	CA	MDD _treated since min.6w_	Severe _average_	MDD	BDI – HAM-D_21_	PD and/or ago. _(*n*=11)_, social phobia _(*n*=7)_, stress-related disorder _(*n*=5)_, somatoform _(*n*=7)_, OCD _(*n*=2)_	Only MDD	Blood _peri_	BS – PCR _Epigenetic Sequencing Methylation analysis software_	Y	Average	9	GRCh38/hg38 _30,236,071;_ _30,236,083;_ _30,236,088;_ _30,236,090;_ _30,236,101;_ _30,236,120;_ _30,236,125;_ _30,236,141;_ _30,236,156_	_**Reg.:**_ _6071–6156_	1^c^	N
Bakusic, 2020	138	45.4	56.5	*n/a*	MDD	Moderate _average_	MDD	HAM-D_17_	*n/a*	MDD + Healthy	Blood _peri_	Pyroseq. _PyroMark Q24_	Y	Average _all + specific sites_	5 units / 23 CpG	GRCh38/hg38 _GenBank NG_011747_	_6101;_ _6088;_ _6101/071_	3	N
Shi, 2017	96	31 _med._ _(21–47)_	60.3	CHA	MDD	Severe _all_	MDD	HAM-D_24_	*n/a*	Only MDD	Blood _peri_	Pyroseq. _Pyro Q-CpG_	Y	Single	6	GRCh37/hg19 _28,562,957;_ _28,562,970;_ _28,562,974;_ _28,562,986;_ _28,562,991;_ _28,562,993_	_5975; 5973;_ _5968; 5956;_ _5952; 5939_	6	N
Iga, 2016	28	45	71.4	JA	MDD	Moderate _average_	MDD	HAM-D_17_	Diabetes, hypertension, gastritis	Only MDD	WBC	Pyroseq. _PyroMark Q24_	Y	Single	9	GRCh37/hg19 _GenBank NC_000017.10_	_6156; 6141;_ _6125; 6120;_ _6101; 6090;_ _6088; 6083;_ _6071_	9	Y
Duman, 2015	67	28.5^a^	0	CA	Depr. Sympt.	Mild _average_	-	BDI-II	-	Only MDD	WBC	MALDI-based _Sequenom Epityper_	N	Average _all + per factor_	3 units / 26 CpG	*n/a*	_**Reg.:**_ _5270–_ _6268_ _3 factors_	3	Y
Lei, 2015	99	48.3	100	AFA	Depr. Sympt.	Mild _average_	-	CES-D	*n/a*	Healthy	Blood _peri_	Microarray _Illumina 450K Human Methylation Beadchip_	N _not in this case_	Average	7 units	*n/a* _Cg27569822,_ _Cg10901968,_ _Cg26741280,_ _Cg25725890,_ _Cg05016953,_ _Cg14692377,_ _Cg03363743_	_6139/090 +_ _6120/071 +_ _6101/50 +_ _6037/5 988 +_ _5796/47 +_ _5668/19 +_ _5457/08_	1	N
van der Knaap, 2015	939	16.2	51.7	Dutch	Depr. Sympt..	*n/a*	-	YSR	Internalizing, Anx., Depr.	Healthy	Blood _peri_	MALDI-based _Agena_	N	Average	11 units	GRCh37/hg19 _**Reg.:**_ _28,562,388–_ _28,563,186_	_**Reg:**_ _5370–_ _6168_	1	N
Okada, 2014	50	40.3	46	JA	MDD	Moderate _all_	MDD	HAM-D_17_	*n/a*	Only MDD	Blood _peri_	MALDI-based _Agena_	N	Average _per unit_	29 units/47 CpG	GRCh37/hg19 _**Reg**.:_ _28,562,388–_ _28,563,186_	spe. CpG sites: *n/a*_**Reg:**_ _5370–_ _6168_	29	N
Kang, 2013	108	54.9	76.4	*n/a*	MDD	Moderate _average_	MDD	HAM-D_17_	*n/a*	Only MDD	Blood _peri_	Pyroseq. _Pyro Q-CpG_	Y	Single	7 CpG	*n/a* _−479 to −350 bp relative to the TSS;_	_6141; 6125;_ _6101; 6090;_ _6088; 6083;_ _6071_	7	N

*Ago.* Agoraphobia, *AFA* African-American, *Anx.* Anxiety, *BD* Bipolar Disorder, *BDI* Beck Depression Inventory, *Blood*_*peri*_ peripheral Blood, *BS-PCR* Bisulphite polymerase chain reaction, *CA* Caucasian, *CES-D* Centre for Epidemiological Studies-Depression scale (CES-D), *CHA* Chinese Han, *CIDI* Composite International Diagnostic Interview, *CpG* Cytosine phosphate guanine, *Depr.* Depression, *EUR* European ancestry, *HA* Hispanic, *HAM-D* Hamilton Depression Rating Scale, *JA* Japanese, *KA* Korean, *MADRS* Montgomery-Åsberg Depression Rating Scale, *MALDI-based* Matrix-Assisted Laser Desorption/Ionisation-based, *MDD* Major Depressive Disorder, MINI: Mini International Neuropsychiatric Interview, N:No, *n/a*: Not available, OCD: Obsessive-Compulsive Disorder, PD: Panic Disorder, PSD: Post Stroke Depression, PTSD: Post-Traumatic Stress Disorder, *RWD* Rwandan, *SCID-I* Structured Clinical Interview for DSM-5, *SCZ* Schizophrenia, *Sympt.* Symptoms, *SLE* Systemic lupus erythematosus, *TSS* Transcription Start Site, *WBC* White Blood Cell, *Y* Yes, *YSR* Youth Self-Report.

^a^Age of the full sample reported in the study.

^b^Not the same sample size for all computed effect sizes.

^c^Multiple questionnaires were used to evaluated depression severity thus effects were first pooled per CpG site/unit – only average effects are reported.

^d^Region analysed in the promoter but not in the 799bp CpG island.

Fifteen studies included patients with Major Depressive Disorder (MDD) as their primary disorder, two used both MDD patients and individuals with depressive symptoms, four included patients with MDD as a comorbid disorder, two utilized healthy individuals with depressive symptoms, and one did not select participants based on depressive symptoms or disorder (Table 2). The severity of depressive symptoms ranged from mild to severe. For the meta-analysis with group comparison, ten studies used healthy controls, one panic disorder patient without depression, and one systemic lupus erythematosus without depression (Table 2). Methylation was assessed in whole blood (k = 16), white blood cells (k = 5), a combination of both (k = 1), capillary dried blood spots (k = 1), or saliva (k = 1). The methods used included bisulphite conversion (k = 6), pyrosequencing (k = 11), MALDI-TOF (k = 5), or microarray analyses (k = 2) (Table 2). Genomic locations were standardized to a common reference assembly (GRCh38/hg38) using the NCBI nucleotide database, Ensembl, and the Genome Browser (Table 2). The assessed CpG sites were located within the promoter region between chr17: 30 235 270 and chr17: 30 237 150, with the most frequently analyzed sites situated within a 799 bp CpG island. Notably, the most commonly tested sites included chr17: 30 236 071, chr17: 30 236 083, chr17: 30 236 088, and chr17: 30 236 090 (Table 2). Among all included studies, two measured methylation at sites other than the 799 bp CpG island on the *SLC6A4* gene promoter. These alternative sites included the AluJb fragment and a region slightly upstream of the CpG Island (Table 2).

**Fig. 2:**
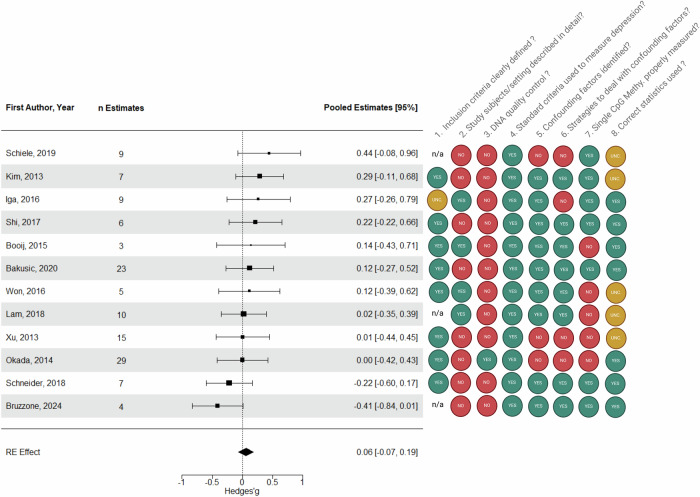
Study-level forest plot of the meta-analysis assessing depression occurrence and *5-HTT* promoter methylation and rating of bias of the included studies according to the checklist for analytical cross-sectional studies (refer to Section 2.4). UNC unclear, n/a not applicable. Notably, Philibert [9] was exclusively incorporated within the systematic review section of this report and thus is not represented in this figure. Nonetheless, its bias ratings are as follows: 1. n/a, 2. NO, 3. NO, 4. YES, 5. NO, 6. NO, 7. NO, 8. UNCLEAR.

Finally, the bias ratings are presented in Figs. 2, 4. As clarified in the methodology section, the bias ratings were directed towards the focal outcomes. However, these outcomes often held a secondary status, thereby increasing the potential for bias in the reported effects. As depicted in Figs. 2, 4, none of the studies fulfilled all the criteria. The most commonly unmet criteria pertained to “exposure measurement” (in our case, unreported DNA extraction method and quality control), description of subjects/settings (with comorbidities, medication and ethnicity frequently missing), and outcome measurement (where we focused on whether the method allowed for single CpG analysis, whether results were reported for each CpG site, and whether genomic locations were clearly and precisely specified). One can also note that the choice of statistical tests was sometimes unclear due to a lack of information on data distribution. In contrast, the criterion evaluating “condition measurement” was consistently satisfied, as all studies employed validated questionnaires or interviews to assess depressive symptoms or depression.

### Depression occurrence and *5-HTT* promoter methylation

#### **Main analysis**

**Fig. 3:**
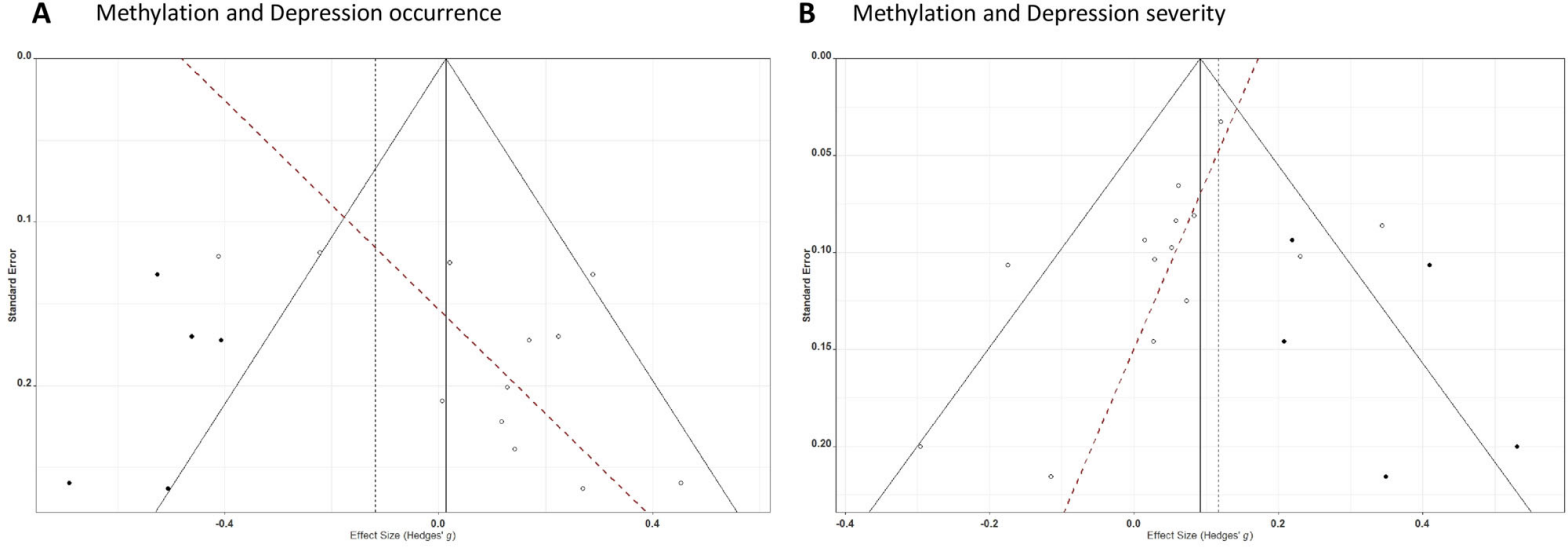
Funnel plots from both meta-analyses. **A** Study-level funnel plot of the meta-analysis assessing depression occurrence and *5-HTT* promoter methylation. **B** Study-level funnel plot of the meta-analysis assessing depression severity and *5-HTT* promoter methylation. The dashed red line represents a regression line connecting the standard error and the effect size. The white dots indicate the combined effects at the study level. The black dots represent potential missing studies, estimated through trim-and-fill analysis.

Overall, twelve studies reporting 127 effect sizes were available for the analysis. The average effect size for depression occurrence and *5-HTT* promoter methylation across the twelve studies was not significant (*p* = 0.362) and almost null (Hedges’ g = 0.06, 95% confidence interval (CI): −0.07 to 0.19; Fig. 2B), with moderate heterogeneity within studies (τ^2^_(2)_ = 0.068, I^2^_(2)_ = 58.0%) and low heterogeneity between studies (τ^2^_(3)_ = 0.020, I^2^_(3)_ = 16.7%). According to the prediction interval, in the universe of populations represented by the included studies, the true effect, in 95% of cases, will fall between moderate negative and moderate to large positive (g = −0.54 to 0.66). A visual asymmetry was assumed (Fig. 3) and confirmed by Egger’s test (intercept = −0.48, *p* = 0.024). The trim and fill analysis suggested five missing studies on the left side (Fig. 3A), providing a small negative adjusted Hedges’ g (g = −0.09, 95% CI: −0.25 to 0.06).

#### **Moderator analysis**

Due to the minimal heterogeneity between studies, a between-study moderator analysis was not conducted. However, as within-study heterogeneity was moderate (I^2^_(2)_ = 58.0%), an analysis was performed to assess the independent effects of individual CpG sites. To do so, only studies that examined single CpG sites were retained in the analysis (Table 2). Furthermore, given the large number of CpG sites tested, many of which were assessed only once, only those evaluated at least three times were included. This filtering reduced the dataset to 7 studies and 33 effects, decreasing the I^2^_(2)_ to 32.5%. No significant differences were detected between CpG sites (Q_M_ = 2.19, df_1_ = 7, df_2_ = 25, *p* = 0.070). According to the GRCh38/hg38 genome assembly references, the results were as follows: ch17: 30 235 939 (k = 3, g = −0.11, 95% CI −0.44 to 0.22), ch17: 30 236 071 (k = 5, g = 0.13, 95% CI −0.23 to 0.49), ch17: 30 236 083 (k = 5, g = 0.27, 95% CI −0.09 to 0.63), ch17: 30 236 088 (k = 5, g = 0.48, 95% CI 0.12 to 0.85), ch17: 30 236 101 (k = 4, g = 0.23, 95% CI −0.13 to 0.58), ch17: 30 236 125 (k = 3, g = 0.44, 95% CI 0.03 to 0.85) and ch17: 30 236 141 (k = 3, g = 0.39, 95% CI −0.02 to 0.79).

### Depression severity and *5-HTT* promoter methylation

#### **Main analysis**

**Fig. 4:**
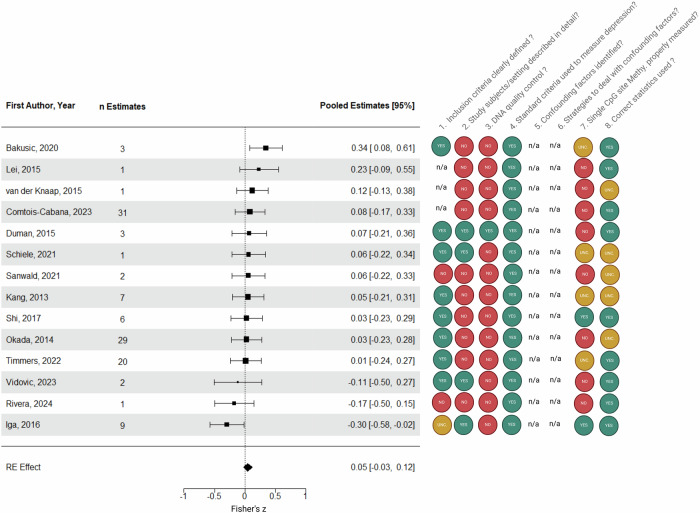
Study-level forest plot of the meta-analysis assessing depression severity and *5-HTT* promoter methylation and rating of bias of the included studies according to the checklist for analytical cross-sectional studies (refer to Section 2.4). UNC unclear, n/a not applicable. Moon [44] was exclusively incorporated within the systematic review section of this report and thus is not represented in this figure. Nonetheless, its bias ratings are as follows: 1. YES, 2. YES, 3. NO, 4. YES, 5. n/a, 6. n/a, 7.YES, 8. YES.

Overall, fourteen studies reporting 116 effect sizes were available for analysis. The average effect size for depression occurrence and *5-HTT* promoter methylation across the fourteen studies was null (Z = 0.05, 95% CI: −0.03 to 0.12, *p* = 0.226; Fig. 3B and Fig. 4), with no heterogeneity between effects within studies (τ^2^_(2)_ = 0.000, I^2^_(2)_ = 0%) and moderate heterogeneity between studies (τ^2^_(3)_ = 0.016, I^2^_(3)_ = 62.4%). According to the prediction interval, in the universe of populations represented by the included studies, the true effect, in 95% of cases, will fall between small negative and small to moderate positive (Z = −0.22 to 0.31). A small visual asymmetry was assumed and confirmed by Egger’s test (intercept = 0.17, *p* = 0.067). The trim and fill analysis suggested five missing studies on the right side (Fig. 3B), providing a slightly bigger adjusted Hedges’ g (g = 0.12, 95% CI: 0.05 to 0.19).

#### **Moderator analysis**

##### **Depression severity – subgroup analysis**

No group differences were detected between depressive symptoms, but no diagnosed depression (k = 1, g = 0.12, 95% CI −0.13 to 0.38), mild (k = 5, Z = 0.05, 95% CI −0.08 to 0.17), moderate (k = 4, Z = 0.04, 95% CI −0.09 to 0.17), moderate to severe (k = 2, Z = 0.01, 95% CI −0.22 to 0.22), and severe (k = 2, Z = 0.04, 95% CI −0.15 to 0.23) depression (Q_M_ = 1.97, df = 4, *p* = 0.853). Yet, one can note that most of the categories were underrepresented.

##### **Methylation techniques – subgroup analysis**

No group differences were detected between bisulfite sequencing (k = 3, Z = 0.03, 95% CI −0.13 to 0.21), MALDI-TOF (k = 4, Z = 0.07, 95% CI −0.06 to 0.20), microarray (k = 2, Z = 0.03, 95% CI −0.20 to 0.26), and pyrosequencing techniques (k = 5, Z = 0.03, 95% CI −0.08 to 0.15) (Q_M_ = 1.66, df = 3, *p* = 0.798).

##### **Cell type – subgroup analysis**

No group differences in depression occurrence and *5-HTT* promoter methylation were detected between peripheral whole blood (k = 11, Z = 0.07, 95% CI −0.01 to 0.15), white blood cells (k = 2, Z = −0.12, 95% CI −0.31 to 0.08) and saliva (k = 1, Z = 0.08, 95% CI −0.17 to 0.33) used for the methylation analysis (Q_M_ = 4.38, df = 2, *p* = 0.224).

##### **Ethnicity**

Five studies were excluded from the analysis as they did not report this information. To maintain sufficient statistical power, ethnic backgrounds were reclassified into broader categories. Specifically, studies with individuals of Caucasian (k = 3) and Dutch (k = 1) descent were grouped under European ancestry; Chinese Han (k = 1) and Japanese (k = 2) under Asian ancestry; and African-American (k = 2) and Rwandan (k = 1) under African-American or African ancestry. No significant differences in depression occurrence or *5-HTT* promoter methylation were observed between Asian ancestry (k = 3, Z = −0.07, 95% CI: −0.22 to 0.08), European ancestry (k = 4, Z = 0.0, 95% CI: −0.09 to 0.20), and African-American or African ancestry (k = 3, Z = 0.03, 95% CI: −0.20 to 0.26).

##### **Age – meta-regression**

Meta-regression revealed that the average sample age was not a significant moderator of the association between depression occurrence and *5-HTT* promoter methylation (k = 14, R^2^ = 0%, *p* = 0.949).

##### **Female percentage – meta-regression**

Meta-regression revealed that the female percentage in the tested sample was not a significant moderator of the association between depression occurrence and *5-HTT* promoter methylation (k = 14, R^2^ = 0%, *p* = 0.850).

## Discussion

This systematic review investigates the association between 5-HTT promoter region methylation levels and depressive symptoms in humans. Two meta-analyses assessing (1) *5-HTT* promoter methylation levels between depressed and non-depressed conditions and (2) the association between *5-HTT* methylation and the severity of depressive symptoms were conducted. A total of 24 studies were encompassed in the systematic review, with two studies lacking adequate data for inclusion in the meta-analyses.

The results of the first meta-analysis, comparing depression occurrence and *5-HTT* promoter methylation (12 studies – 2 028 subjects – 127 effects), found no significant effect (g = 0.06, 95% CI −0.01 to 0.21). An asymmetry was detected, leading to a very small negative average effect size upon adjustment (g = −0.09, 95% CI: −0.14 to 0.11). The heterogeneity within-study was moderate (I^2^_(2)_), while the between-study heterogeneity was low (I^2^_(3)_), impairing the possibility to test for moderators at study-level.The overall heterogeneity, indexed by the prediction interval, indicates that the true effect falls between moderate negative and moderate to large positive in the universe of populations represented by the included studies. The moderator analysis assessing the effect of single CpG sites at effect size level was not significant. However, this analysis was conducted on a substantially smaller subset of the dataset due to limitations in data availability and comparability. In the second meta-analysis, focusing on the association between depression severity and *5-HTT* promoter methylation (14 studies – 2 296 subjects - 116 effects), the results revealed a null average effect size (Z = 0.05, 95% CI −0.03 to 0.16). An asymmetry was also detected, leading to a small positive average effect size upon adjustment (g = 0.12, 95% CI: 0.05 to 0.19). Heterogeneity within individual studies was null, while between-study heterogeneity was moderate. The prediction interval indicated a range for the true effect from small negative to small/moderate positive. No significant moderators were identified. Among the comprehensive array of studies incorporated, it’s notable that all reported effects carried the potential for bias.

Our results suggest that depressed individuals exhibit no obvious difference in *5-HTT* promoter methylation levels compared to non-depressed individuals. This observation holds true even after adjusting for publication bias, indicated by the asymmetry of these effects. The adjusted effect following post-trim-and-fill analysis was even rendered slightly negative. Nevertheless, this outcome does not inherently dismiss the potential link between depressive disorder and *5-HTT* promoter methylation, especially when considering the notable overall heterogeneity of the results (as reflected by the prediction interval). For example, the moderate within-study heterogeneity observed in meta-analysis (1) (may also exist in meta-analysis (2) but completely overlapped by sampling error), suggests effects may vary at the level of single CpG sites or CpG units across different studies. The CpG site moderator analysis conducted in meta-analysis (1) did not provide significant evidence to support this hypothesis (*p* = 0.070). However, this analysis included only 26% of the available effects (33 out of 127) and only 8 of the 69 CpG sites reported in meta-analysis (1). Despite this, we still observed variability in the average effect between the tested CpGs (ranging from −0.11 to 0.48). Having more reports of all single-CpG sites in the *5-HTT* promoter (with their precise locations on the used genome assembly) would help provide more conclusive evidence. The influence of specific CpG sites (such as those located on the promoter or near transcription factor binding sites) on gene transcription could be substantial. This phenomenon can be elucidated from a physical standpoint, as methylation at a given CpG site has the capacity to influence DNA accessibility to transcription machinery, the interaction of regulatory proteins, the structure of chromatin, and interconnections within various genomic segments [54, 55]. Together, those factors collectively determine gene expression. Further studies may consider combining single-CpG technics covering the full *5-HTT* promoter with other OMICS approaches in longitudinal depressive research to be able to pinpoint the most influential CpG-sites.

Despite the lack of success in testing for moderators, one can nonetheless consider their theoretical implications and evaluate the progress made thus far. First, there were inherent limitations in the analysis parameters applied to the tested moderators in meta-analysis 2. While a diverse array of effects was detected, justifying the application of a three-level meta-analysis, the limited number of included studies led to skewed moderator categories distribution, limiting the extent of their analysis. Also, the classification of certain moderators, such as depression severity and age, was predominantly based on average values rather than patient-specific data, introducing potential approximations.

Second, a variety of methylation analysis techniques have been employed; however, not all of them facilitate single-CpG analysis. Even when this analysis was feasible, articles didn’t consistently report all scores. For example, targeted bisulfite sequencing, particularly when combined with next-generation sequencing (NGS), enables high-resolution quantification of DNA methylation at specific CpG sites with high sensitivity. In contrast, MALDI-TOF, though capable of detecting methylation changes, is generally less quantitative and does not provide single-nucleotide resolution, making it less reliable for precise CpG-site analysis. For microarrays, results depend on probe design and hybridization signals. Although some platforms, such as Illumina arrays, use a Single Base Extension approach to distinguish methylated and unmethylated cytosines at specific CpG sites (e.g., based on different dyes), the effectiveness of this method depends on probe coverage and design. In our analysis, the two studies using microarrays did not provide sufficient single-site resolution. Pyrosequencing has traditionally been used for targeted methylation analysis, but its lower throughput and limited scalability make it less suitable for larger studies. Targeted bisulfite sequencing with NGS should be prioritized for single-gene methylation analysis due to its high accuracy, single-nucleotide resolution, and ability to capture CpG-specific methylation patterns across the entire gene of interest. However, the results of the moderator analysis in the second meta-analysis, which was based on the aggregated methylation scores, did not indicate significant differences.

Third, even in the second meta-analysis, it was impossible to test all moderators’ extent fully. For example, when considering sample type, only peripheral blood, white blood cells and saliva were reported. Nevertheless, multiple studies have demonstrated that gene methylation status doesn’t correlate perfectly across tissues [56–58], even within the same cell categories (e.g., leukocytes) [59, 60]. Consequently, relying solely on peripheral blood or white blood cells may result in an amalgamation of cell-specific methylation statuses, potentially skewing the overall interpretation. Furthermore, *5-HTT* is prominently expressed in platelets [61] but less so in other peripheral cells. Consequently, comparing peripheral blood and white blood samples could introduce further complexity. Other peripheral cells in which *5-HTT* is strongly expressed include enterochromaffin and intestinal epithelial cells located in the mucosa of the stomach and intestines. Exploring these cells could indirectly assess the link between *5-HTT* promoter methylation and depression. Moving beyond peripheral cells, neural cells from the raphe nuclei (serotonergic nuclei), the pre-frontal cortex, the amygdala, or the hippocampus (brain regions substantially affected in depressed individuals) [62, 63] could provide more direct insights into evaluating the association between the methylation status of serotonergic genes and their connection to depressive symptoms. However, obtaining these neural cells from individuals in a quiescent state (without the potential for event-induced acute methylation) and minimizing temporal discrepancies with depressive assessments presents substantial challenges. By successfully overcoming these challenges and conducting cell-specific methylation analysis, we would not only gain a direct perspective on this association but also address the question of whether *5-HTT* methylation in peripheral or white blood cells can serve as a reliable comparison to that in central tissues (or where *5-HTT* is highly expressed). This development may eventually pave the way for implementing such analyses into routine medical testing as a standardized diagnostic measure for disorders or treatment progression.

Fourth, gene expression was very inconsistently assessed. Only four studies examined the *5-HTT* expression level, while most studies assumed that high *5-HTT* promoter methylation leads to reduced expression—a simplification that might not hold true for the sample types studied. Moreover, it is essential to recognize that other epigenetic mechanisms, such as histone modifications (acetylation, methylation, phosphorylation, and ubiquitination), non-coding RNAs, or chromatin remodeling can alter gene expression. Therefore, it is of major importance to assess methylation and gene expression to conclude the association between *5-HTT* and depressive symptoms. To have a complete picture of the epigenetic drive of depression, one should also consider assessing the subsequent proteins of interest. As mentioned earlier, both methylation and gene expression are processes that can vary between cells, and it remains to be established whether changes for 5-HTT in the periphery are equivalent to those in the central nervous system. Thus, to overcome the indirectness of peripheral measures, a particularly interesting study by Bruzzone and colleagues [49] used imaging data (i.e., Positron Emission Tomography and Magnetic Resonance) to assess the interaction between epigenetic changes and neurological markers of the 5-HTT gene (together with the post-synaptic serotonin receptor 5-HT_4_). However, no statistically significant association was found between the CpG sites (chr17: 30 236 071, chr17: 30 236 083, chr17: 30 236 088, and chr17: 30 236 090) they measured and markers of serotonergic neurotransmission in patients with MDD or healthy controls. Nonetheless, further studies using multi-level assessments of a biological machinery, such as the approach used here, are needed to better understand the central effect of epigenetic changes that may influence behavior. Fifth, some relevant moderators were not consistently reported in the studies, hindering their analysis. Indeed, factors potentially influencing the *5-HTT* promoter methylation status, such as genetic (depression inheritance), stressful life events (in particular during childhood), or metabolic factors (e.g., BMI leading to increased chronic inflammation eventually influencing methylation), remain unaccounted for. While comorbidity and medication were occasionally reported (as indicated by item 2 of the bias rating tool), they were often mixed within the same sample. Variability in those parameters may have contributed to increased sampling error and heterogeneity in our results. Future studies could benefit from recruiting less heterogeneous samples to yield clearer results. Notably, existing literature has shown that pharmacological interventions can potentially alter the methylation status of the *5-HTT* gene [14, 23, 31, 64], emphasizing the importance of considering medication variability in patient recruitment. Another potential moderator, ethnicity, was inconsistently reported, with data missing in 7 out of the 24 studies. Given the influence of ethnicity on epigenetic markers [65], this parameter should be reported more systematically even if, in our case, no significant effects were detected.

Lastly, the potential for approximation should be acknowledged, as it could hinder the detection of effects due to the diverse biological underpinnings of depression and its various markers. For example, the findings of Zhao and colleagues, who explored methylation variations and depressive symptoms among twins, suggest that genetic disparities play a significant role when evaluating *5-HTT* methylation and depressive symptoms [66]. Already within the *5-HTT* gene, genetic variants (e.g., *5-HTTLPR*, *STin2*) might moderate the association between its methylation and depressive symptoms, as demonstrated by Iga et al. [22] and Kim et al. [8]. Additionally, other markers such as BDNF, kynurenine branch, inflammation, serotonin, dopamine, and norepinephrine have all been linked to depression, yet their involvement varies depending on depressive phenotypes [10, 67]. This variability is evident in the context of selective serotonin reuptake inhibitors, where not all patients experience remission. This is not necessarily because the medication lacks the anticipated physiological effect but rather because serotonin may not be the sole underlying issue. Epigenetic changes in other genes associated with the regulation of the previously mentioned biomarkers may also be linked to depression and could interact with *5-HTT*. Studies employing an epigenome-wide approach could help assess this factor and uncover the epigenetic footprint of psychiatric disorders.

Compiling these limitations, we can establish a set of guidelines for future research in this field. (1) Researchers should strive to integrate methylation data with other OMICS approaches, including transcription and, whenever feasible, protein level assessments. (2) Preference should be given to single-site methylation techniques over global measures. (3) It is crucial to provide detailed reports of CpG site locations, including precise genomic coordinates and the reference genome assembly used, to ensure standardization and reproducibility across studies. (4) Methylation across different cell types should not be considered equal, prompting a focus on single-cell type analyses, particularly in cell types where *5-HTT* is highly expressed, both peripherally and centrally. (5) Methodological descriptions should be as comprehensive as possible to enable experiment reproducibility, encompassing aspects such as DNA extraction, quality-control, and detailed methylation techniques, among others. (6) A minimal time gap should be aimed between depression assessment and the collection of biological samples. (7) The testing of homogeneous patient groups is recommended to exclude potential influences of other factors recognized to affect epigenetic processes (e.g., age). (8) Comprehensive descriptions of patient characteristics should be provided, without neglecting aspects known to affect both methylation and the development of depression (e.g., medication, childhood trauma).

## Conclusion

In conclusion, our meta-analysis reveals a lack of conclusive evidence regarding the associations between *5-HTT* promoter methylation and the occurrence or severity of depression. No moderators of the results were identified at the effect-size level (for meta-analysis (1)) or at the study level (for meta-analysis (2)). However, the limited within- and between-study heterogeneity, along with the availability, reliability, and comparability of the data used for moderator analysis, highlights the need for further research on this topic to draw definitive conclusions, in particular for single CpG sites where variability in the results has been shown. The current studies have contributed to an initial understanding of how epigenetics could influence behavioral outcomes. However, it is imperative for future research to delve deeper, focusing on cell-specific and site-specific CpG methylation in more homogeneous sample populations while incorporating multi-level assessments. This entails not only examining methylation patterns but also investigating gene expression and protein levels. Furthermore, neglecting other genes linked to depression may hinder a comprehensive grasp of the broader epigenetic changes induced by depressive disorders, potentially uncovering a primary driving factor.

## Supplementary information

Supplementary Material A

Supplementary Material B
