# Supplementary material for: Exploring the association between serotonin transporter promoter region methylation levels and depressive symptoms: a systematic review and multi-level meta-analysis
Source: Transl Psychiatry. 2025 May 3;15:161. doi: 10.1038/s41398-025-03356-w (PMC12049537; doi:10.1038/s41398-025-03356-w)
Supplement: Supplementary file 2 — Supplementary Material B [file 41398_2025_3356_MOESM2_ESM.pdf]

Exploring the Association Between Serotonin Transporter Promoter Region Methylation and Depressive Symptoms: A Systematic Review and Multi-level meta-analysis

Dr. Florian JAVELLE

15/02/2025

Contents

1. Introduction **1**

2. Depression occurence and 5-HTT promoter methylation **2**

2.1 Main analysis . . . 2

2.1.1 Prepare the workspace . . . 2

2.1.2 Charge the dataset . . . 2

2.1.3 Three-level meta-analysis . . . 3

2.2 Moderator analysis . . . 13

3. Depression severity and 5-HTT promoter methylation **16**

3.1 Main analysis . . . 16

3.1.1 Charge the dataset . . . 16

3.1.2 Three-level meta-analysis . . . 17

3.2 Moderator analysis . . . 27

3.2.1 Categorical moderators . . . 27

3.2.2 Continuous moderators . . . 29

4. Conclusion **31**

1. Introduction

This systematic review and multi-level meta-analysis aims to examine the association between 5-HTT promoter region methylation levels and depressive symptoms in humans. Using this R markdown report, we hope to help the interested reader understand the analyses performed and the R script used. To keep this report easy to understand, we have not printed the longest script sections (e.g., functions creation). Nevertheless, the complete script, the datasets (depression occurrence and depression severity), and the computation tables are available on Open Science Framework (https://osf.io/guatx/?view_only=de7aafa4cad848ca97ab052086b7bf10).

Our article comprehends two meta-analyses assessing 1) 5-HTT promoter methylation levels between depressed and non-depressed conditions and 2) the association between 5-HTT methylation and the intensity of depressive symptoms. Please note that the methods detailing the complete analysis are reported in the main article but not in this report.

2. Depression occurence and 5-HTT promoter methylation

2.1 Main analysis

2.1.1 Prepare the workspace

First, we have to install and charge the necessary libraries for the analyses.

```
     # To install and charge the libraries
if(!require(meta)) install.packages("meta", repos = "http://cran.us.r-project.org")
if(!require(metafor)) install.packages("metafor", repos = "http://cran.us.r-project.org")
if(!require(metaviz)) install.packages("metaviz", repos = "http://cran.us.r-project.org")
if(!require(tidyr)) install.packages("tidyr", repos = "http://cran.us.r-project.org")
if(!require(data.table)) install.packages("data.table", repos = "http://cran.us.r-project.org")
if(!require(dplyr)) install.packages("dplyr", repos = "http://cran.us.r-project.org")
if(!require(stringr)) install.packages("stringr", repos = "http://cran.us.r-project.org")
if(!require(readr)) install.packages("readr", repos = "http://cran.us.r-project.org")
if(!require(ggplot2)) install.packages("ggplot2", repos = "http://cran.us.r-project.org")
if(!require(reshape2)) install.packages("reshape2", repos = "http://cran.us.r-project.org")
if(!require(cowplot)) install.packages("cowplot", repos = "http://cran.us.r-project.org")
if(!require(gridExtra)) install.packages("gridExtra", repos = "http://cran.us.r-project.org")
if(!require(forestplot)) install.packages("foresplot", repos = "http://cran.us.r-project.org")
if(!require(metaSEM)) install.packages("metaSEM", repos = "http://cran.us.r-project.org")
if(!require(OpenMx)) install.packages("OpenMx", repos = "http://cran.us.r-project.org")

library(OpenMx)
library(meta)
library(ggplot2)
library(metafor)
library(metaviz)
library(tidyr)
library(dplyr)
library(data.table)
library(stringr)
library(readr)
library(reshape2)
library(cowplot)
library(gridExtra)
library(forestplot)
library(metaSEM)
```

2.1.2 Charge the dataset

Let's now charge the dataset from local storage. We are going to call it "fullData" and ensure that all variables are properly considered (e.g., numeric, character). We are also going to proceed to some sanity checks such as checking the number of studies and outcomes (creating an outcome ID is also useful for later visualisation purposes). Finally, we are going to compute the number of outcomes per study and keep it in our dataset.

```
     # To upload the file from local storage
fullData<-read.csv("C:/Users/Florian/Downloads/5HTT_group_comparison_nocorrection_FINAL.csv",
                   sep=";", dec=".")

     # To be sure that our values are considered as numeric values
fullData$g<- as.numeric(fullData$g)
fullData$V_g<- as.numeric(fullData$V_g)
```

```r
fullData$SE_g<- as.numeric(fullData$SE_g)
fullData$Age<- as.numeric(fullData$Age)
fullData$Perc_fem<- as.numeric(fullData$Perc_fem)

     # Some sanity checks
unique_studies  <- unique(fullData$Study_ID)  # unique studies
nr_studies  <- length(unique_studies)
nr_studies
```

```
## [1] 12
```

```r
     # Add an outcome ID for later multilevel modeling
fullData$Outcome_ID <- seq(from = 1, to = nrow(fullData), by = 1)

     # Add n_effects (number of effects per study) for later visualisation purposes
fullData<-fullData %>% group_by(Study_ID)%>% mutate(n_effects=n())
```

2.1.3 Three-level meta-analysis

Let's now begin our analysis. The first package that we are going to use is metafor, therefore we have to create a matrix suitable to the functions in this library. In other words, we are going to convert our data frame to an escalc object for the model fit with metafor.

```r
fullData <-escalc(measure = "GEN", yi = g, vi = V_g, data = fullData, ci = ,)
```

Then, we are going to construct the variance/covariance matrix assuming a correlation of rho=0.6 between effects measured concurrently. This rho can be modified based on the type of effects you measure. In our case, literature has shown various levels of association between CpG sites, yet 0.6 is presented as a standard.

```r
V<-vcalc(vi, cluster=Study_ID, obs=Outcome_ID, data=fullData, rho=0.6)
```

Now, we can do the main analysis.

```r
threelevel.metafor<-rma.mv(g, V,
                           random = ~ 1 | Study_ID/Outcome_ID,
                           tdist = TRUE,
                           test= "t",
                           data = fullData,
                           method = "ML",
                           level=95)
summary(threelevel.metafor)
```

```
##
## Multivariate Meta-Analysis Model (k = 127; method: ML)
##
##   logLik  Deviance       AIC       BIC      AICc
## -33.8481  349.9915   73.6962   82.2288   73.8914
##
## Variance Components:
##
##              estim    sqrt  nlvls  fixed               factor
## sigma^2.1   0.0195  0.1398     12     no             Study_ID
## sigma^2.2   0.0682  0.2611    127     no  Study_ID/Outcome_ID
```

```
##
## Test for Heterogeneity:
## Q(df = 126) = 682.6223, p-val < .0001
##
## Model Results:
##
## estimate      se    tval   df    pval    ci.lb   ci.ub
##   0.0590  0.0646  0.9141  126  0.3624  -0.0687  0.1868
##
## ---
## Signif. codes:  0 '***' 0.001 '**' 0.01 '*' 0.05 '.' 0.1 ' ' 1
```

Overall, eleven studies reporting 127 effect sizes are available for the analysis. The average effect size for depression occurrence and 5-HTT promoter methylation across the eleven studies is not significant (p=.085) and very small (Hedges' g = 0.06, 95% confidence interval (CI): -0.07 to 0.19) Let's now compute its prediction interval as index of the overall heterogeneity.

```
predict(threelevel.metafor, intervall="prediction")
```

```
## Warning: Extra argument ('intervall') disregarded.
```

```
##
##    pred     se   ci.lb  ci.ub   pi.lb  pi.ub
##  0.0590 0.0646 -0.0687 0.1868 -0.5408 0.6588
```

According to the prediction interval, in the universe of populations represented by the included studies, the true effect, in 95% of cases, will fall between moderate negative and moderate to large positive (g = -0.54 to 0.66).

Let's create a forest plot to visualise all the effects. We are going to organise it by the size of the effects and put a dotted line between each study to ease the reading.

```
dd <- c(0,diff(fullData$Study_ID))
dd[dd < 0] <- 1
rows <- (1:127) + cumsum(dd)

par(tck=-0.01, mgp = c(1.6,0.2,0), mar=c(3,8,0,6))
forest(threelevel.metafor, cex=0.6, mlab= "RE Effect", header="First Author, Year, Effect",
       shade="zebra",rows = rows, cex.axis = 0.8, cex.lab = 0.8,xlab = "Hedges'g",
       slab=paste(Short_Reference, year, " effect", esid, sep=","), ylim=c(0, max(rows)+3),
       efac=c(0,1),  xlim=c(-3,3), alim=c(-1, 1), at=seq(-1,3, by=1))
abline(h = rows[c(1,diff(rows)) == 2] - 1, lty="dotted")
```

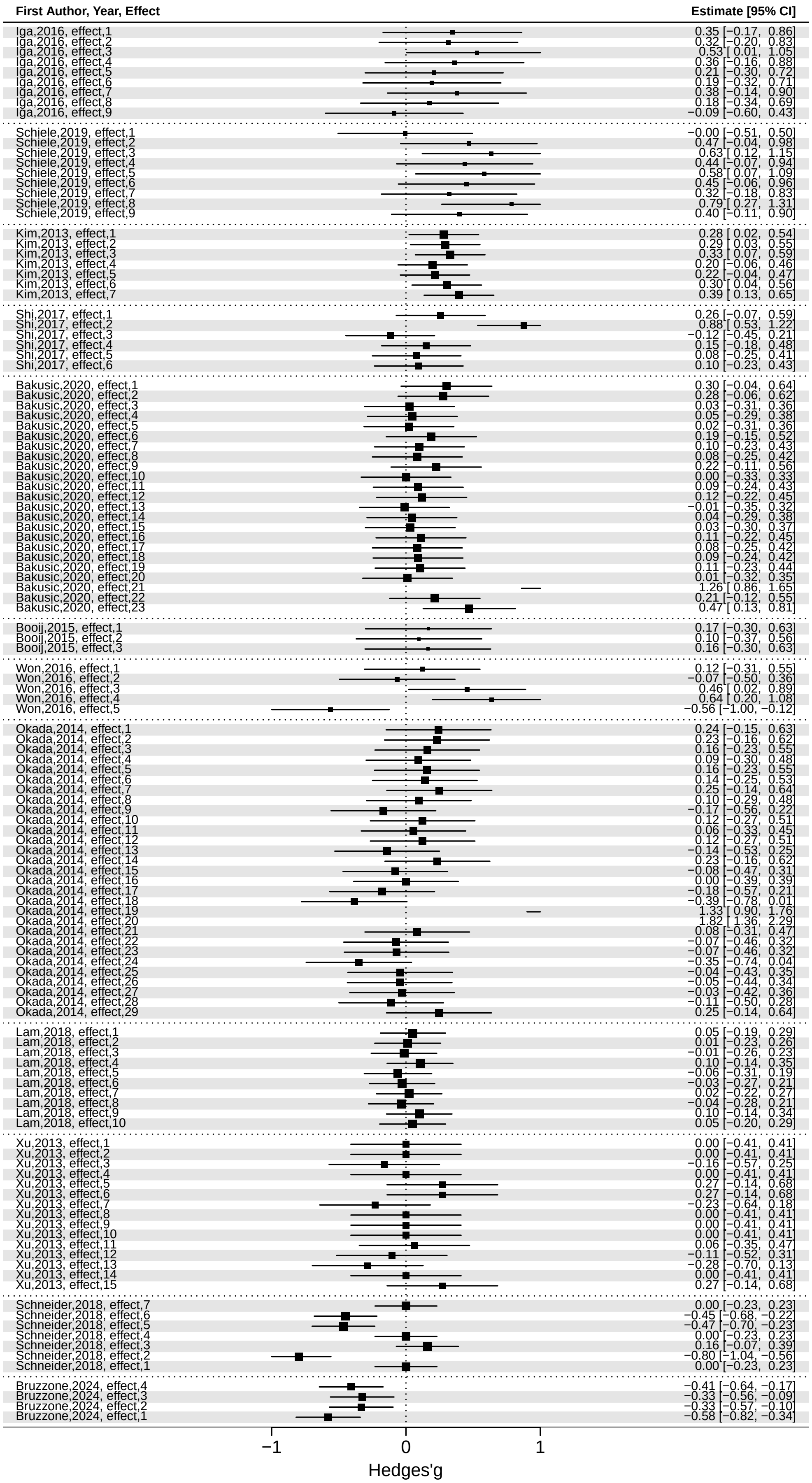
First Author, Year, Effect
Estimate [95% CI]
Iga,2016, effect,1 0.35 [−0.17, 0.86]
Iga,2016, effect,2 0.32 [−0.20, 0.83]
Iga,2016, effect,3 0.53 [0.01, 1.05]
Iga,2016, effect,4 0.36 [−0.16, 0.88]
Iga,2016, effect,5 0.21 [−0.30, 0.72]
Iga,2016, effect,6 0.19 [−0.32, 0.71]
Iga,2016, effect,7 0.38 [−0.14, 0.90]
Iga,2016, effect,8 0.18 [−0.34, 0.69]
Iga,2016, effect,9 −0.09 [−0.60, 0.43]
Schiele,2019, effect,1 −0.00 [−0.51, 0.50]
Schiele,2019, effect,2 0.47 [−0.04, 0.98]
Schiele,2019, effect,3 0.63 [0.12, 1.15]
Schiele,2019, effect,4 0.44 [−0.07, 0.94]
Schiele,2019, effect,5 0.58 [0.07, 1.09]
Schiele,2019, effect,6 0.45 [−0.06, 0.96]
Schiele,2019, effect,7 0.32 [−0.18, 0.83]
Schiele,2019, effect,8 0.79 [0.27, 1.31]
Schiele,2019, effect,9 0.40 [−0.11, 0.90]
Kim,2013, effect,1 0.28 [0.02, 0.54]
Kim,2013, effect,2 0.29 [0.03, 0.55]
Kim,2013, effect,3 0.33 [0.07, 0.59]
Kim,2013, effect,4 0.20 [−0.06, 0.46]
Kim,2013, effect,5 0.22 [−0.04, 0.47]
Kim,2013, effect,6 0.30 [0.04, 0.56]
Kim,2013, effect,7 0.39 [0.13, 0.65]
Shi,2017, effect,1 0.26 [−0.07, 0.59]
Shi,2017, effect,2 0.88 [0.53, 1.22]
Shi,2017, effect,3 −0.12 [−0.45, 0.21]
Shi,2017, effect,4 0.15 [−0.18, 0.48]
Shi,2017, effect,5 0.08 [−0.25, 0.41]
Shi,2017, effect,6 0.10 [−0.23, 0.43]
Bakusic,2020, effect,1 0.30 [−0.04, 0.64]
Bakusic,2020, effect,2 0.28 [−0.06, 0.62]
Bakusic,2020, effect,3 0.03 [−0.31, 0.36]
Bakusic,2020, effect,4 0.05 [−0.29, 0.38]
Bakusic,2020, effect,5 0.02 [−0.31, 0.36]
Bakusic,2020, effect,6 0.19 [−0.15, 0.52]
Bakusic,2020, effect,7 0.10 [−0.23, 0.43]
Bakusic,2020, effect,8 0.08 [−0.25, 0.42]
Bakusic,2020, effect,9 0.22 [−0.11, 0.56]
Bakusic,2020, effect,10 0.00 [−0.33, 0.33]
Bakusic,2020, effect,11 0.09 [−0.24, 0.43]
Bakusic,2020, effect,12 0.12 [−0.22, 0.45]
Bakusic,2020, effect,13 −0.01 [−0.35, 0.32]
Bakusic,2020, effect,14 0.04 [−0.29, 0.38]
Bakusic,2020, effect,15 0.03 [−0.30, 0.37]
Bakusic,2020, effect,16 0.11 [−0.22, 0.45]
Bakusic,2020, effect,17 0.08 [−0.25, 0.42]
Bakusic,2020, effect,18 0.09 [−0.24, 0.42]
Bakusic,2020, effect,19 0.11 [−0.23, 0.44]
Bakusic,2020, effect,20 0.01 [−0.32, 0.35]
Bakusic,2020, effect,21 1.26 [0.86, 1.65]
Bakusic,2020, effect,22 0.21 [−0.12, 0.55]
Bakusic,2020, effect,23 0.47 [0.13, 0.81]
Booij,2015, effect,1 0.17 [−0.30, 0.63]
Booij,2015, effect,2 0.10 [−0.37, 0.56]
Booij,2015, effect,3 0.16 [−0.30, 0.63]
Won,2016, effect,1 0.12 [−0.31, 0.55]
Won,2016, effect,2 −0.07 [−0.50, 0.36]
Won,2016, effect,3 0.46 [0.02, 0.89]
Won,2016, effect,4 0.64 [0.20, 1.08]
Won,2016, effect,5 −0.56 [−1.00, −0.12]
Okada,2014, effect,1 0.24 [−0.15, 0.63]
Okada,2014, effect,2 0.23 [−0.16, 0.62]
Okada,2014, effect,3 0.16 [−0.23, 0.55]
Okada,2014, effect,4 0.09 [−0.30, 0.48]
Okada,2014, effect,5 0.16 [−0.23, 0.55]
Okada,2014, effect,6 0.14 [−0.25, 0.53]
Okada,2014, effect,7 0.25 [−0.14, 0.64]
Okada,2014, effect,8 0.10 [−0.29, 0.48]
Okada,2014, effect,9 −0.17 [−0.56, 0.22]
Okada,2014, effect,10 0.12 [−0.27, 0.51]
Okada,2014, effect,11 0.06 [−0.33, 0.45]
Okada,2014, effect,12 0.12 [−0.27, 0.51]
Okada,2014, effect,13 −0.14 [−0.53, 0.25]
Okada,2014, effect,14 0.23 [−0.16, 0.62]
Okada,2014, effect,15 −0.08 [−0.47, 0.31]
Okada,2014, effect,16 0.00 [−0.39, 0.39]
Okada,2014, effect,17 −0.18 [−0.57, 0.21]
Okada,2014, effect,18 −0.39 [−0.78, 0.01]
Okada,2014, effect,19 1.33 [0.90, 1.76]
Okada,2014, effect,20 1.82 [1.36, 2.29]
Okada,2014, effect,21 0.08 [−0.31, 0.47]
Okada,2014, effect,22 −0.07 [−0.46, 0.32]
Okada,2014, effect,23 −0.07 [−0.46, 0.32]
Okada,2014, effect,24 −0.35 [−0.74, 0.04]
Okada,2014, effect,25 −0.04 [−0.43, 0.35]
Okada,2014, effect,26 −0.05 [−0.44, 0.34]
Okada,2014, effect,27 −0.03 [−0.42, 0.36]
Okada,2014, effect,28 −0.11 [−0.50, 0.28]
Okada,2014, effect,29 0.25 [−0.14, 0.64]
Lam,2018, effect,1 0.05 [−0.19, 0.29]
Lam,2018, effect,2 0.01 [−0.23, 0.26]
Lam,2018, effect,3 −0.01 [−0.26, 0.23]
Lam,2018, effect,4 0.10 [−0.14, 0.35]
Lam,2018, effect,5 −0.06 [−0.31, 0.19]
Lam,2018, effect,6 −0.03 [−0.27, 0.21]
Lam,2018, effect,7 0.02 [−0.22, 0.27]
Lam,2018, effect,8 −0.04 [−0.28, 0.21]
Lam,2018, effect,9 0.10 [−0.14, 0.34]
Lam,2018, effect,10 0.05 [−0.20, 0.29]
Xu,2013, effect,1 0.00 [−0.41, 0.41]
Xu,2013, effect,2 0.00 [−0.41, 0.41]
Xu,2013, effect,3 −0.16 [−0.57, 0.25]
Xu,2013, effect,4 0.00 [−0.41, 0.41]
Xu,2013, effect,5 0.27 [−0.14, 0.68]
Xu,2013, effect,6 0.27 [−0.14, 0.68]
Xu,2013, effect,7 −0.23 [−0.64, 0.18]
Xu,2013, effect,8 0.00 [−0.41, 0.41]
Xu,2013, effect,9 0.00 [−0.41, 0.41]
Xu,2013, effect,10 0.00 [−0.41, 0.41]
Xu,2013, effect,11 0.06 [−0.35, 0.47]
Xu,2013, effect,12 −0.11 [−0.52, 0.31]
Xu,2013, effect,13 −0.28 [−0.70, 0.13]
Xu,2013, effect,14 0.00 [−0.41, 0.41]
Xu,2013, effect,15 0.27 [−0.14, 0.68]
Schneider,2018, effect,7 0.00 [−0.23, 0.23]
Schneider,2018, effect,6 −0.45 [−0.68, −0.22]
Schneider,2018, effect,5 −0.47 [−0.70, −0.23]
Schneider,2018, effect,4 0.00 [−0.23, 0.23]
Schneider,2018, effect,3 0.16 [−0.07, 0.39]
Schneider,2018, effect,2 −0.80 [−1.04, −0.56]
Schneider,2018, effect,1 0.00 [−0.23, 0.23]
Bruzzone,2024, effect,4 −0.41 [−0.64, −0.17]
Bruzzone,2024, effect,3 −0.33 [−0.56, −0.09]
Bruzzone,2024, effect,2 −0.33 [−0.57, −0.10]
Bruzzone,2024, effect,1 −0.58 [−0.82, −0.34]
−1
0
1
Hedges'g

Nice. We can note that we still have the result of the multilevel modeling and that all the effects are not considered in a univariate manner. Furthermore, we can see that within a single study (thus depending on the CpG site) the effects can change significantly. It's quite long though. Let's do a second one, with the aggregated scores per study.

```
agg<-aggregate.escalc(fullData, cluster=Study_ID, V=vcov(threelevel.metafor, type="obs"),
                      addk=TRUE)

res2<-rma(yi, vi, method="EE", data=agg )

forest(res2, xlim=c(-4,5), mlab="RE Effect", slab=paste(Short_Reference, year, sep=", "),
       xlab = "Hedges'g", shade="zebra", header=c("First Author, Year", "Pooled Estimates [95%]"),
       ilab=agg$n_effects, ilab.xpos=-2, order = -res2$yi)
text(-2, res2$k+2, "n Estimates", font=2)
```

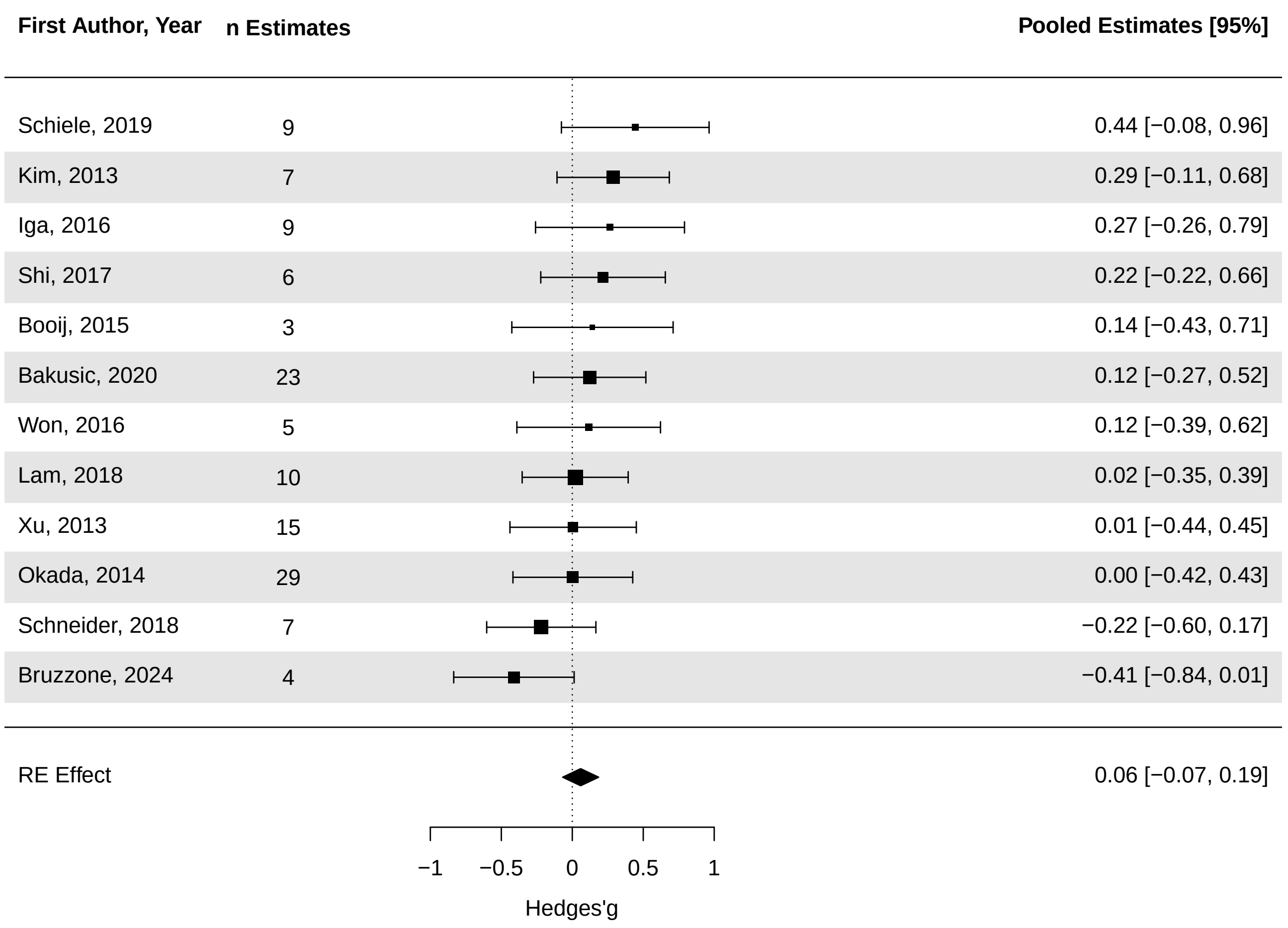

This forest plot is more accessible to navigate, yet it provides only a restricted insight into within-study heterogeneity. Given our previous graph, we are aware that the varying effects within each study primarily contribute to the notable within-study heterogeneity, which is not fully attributed to significant sampling errors. Given the substantial within-study heterogeneity, it seems unlikely that significant between-study heterogeneity will be present. An indicator of this is apparent when observing the overlap among all confidence intervals. I am going to create a function called "var.comp" so that we can assess properly the different levels of heterogeneity. Nevertheless, it is quite long so I will not print it below (refer to the R script).

Hourra! That's done. So let's test it out.

```r
i2<-var.comp(threelevel.metafor)
print(i2)
```

```
## $results
##         % of total variance    I2
## Level 1            25.30783   ---
## Level 2            58.04699 58.05
## Level 3            16.64519 16.65
##
## $totalI2
## [1] 74.69217
##
## $plot
```

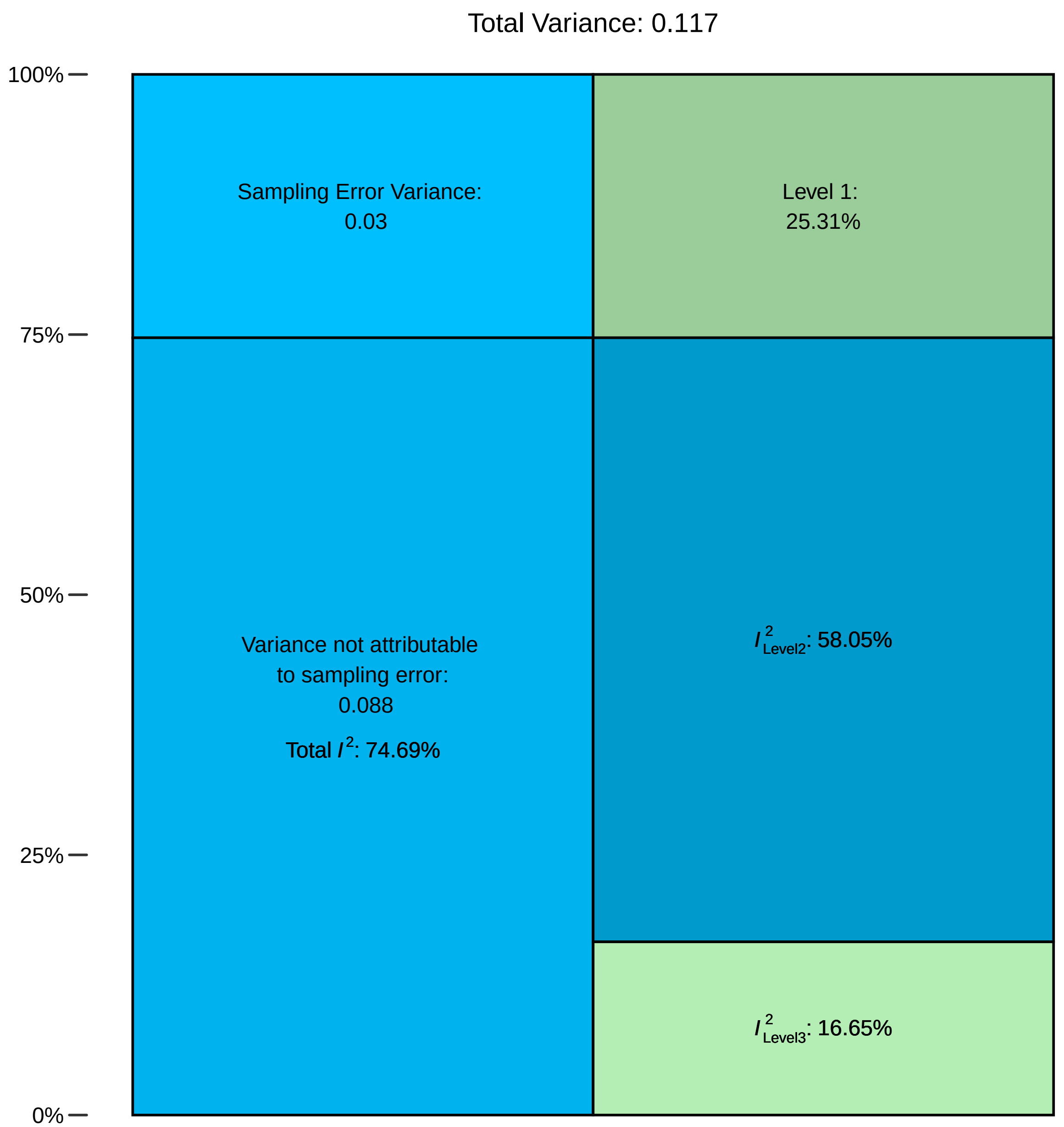

```
##
## attr(,"class")
## [1] "mlm.variance.distribution" "list"
```

Exactly, as planned: The heterogeneity within-studies is moderate (T2(2)=0.068, I2(2)= 58.0%) and the heterogeneity between-studies (T2(3)=0.020, I2(3)= 16.7%) is low. Remark: The T2's were given when we did the main analysis.

Now, while the use of a three-level meta-analysis is justified from a data structure perspective, we can still compare it a two-level model to have a better idea of the fit of the chosen model.

```
twolevel.metafor<-rma.mv(g, V,
                         random = ~ 1 | Study_ID/Outcome_ID,
                         tdist = TRUE,
                         test= "t",
                         data = fullData,
                         method = "ML",
                         sigma2=c(0, NA))

summary(twolevel.metafor)
```

```
##
## Multivariate Meta-Analysis Model (k = 127; method: ML)
##
##   logLik  Deviance       AIC       BIC      AICc
## -34.8216  351.9384   73.6432   79.3315   73.7399
##
## Variance Components:
##
##             estim    sqrt  nlvls  fixed              factor
## sigma^2.1  0.0000  0.0000     12    yes            Study_ID
## sigma^2.2  0.0723  0.2689    127     no  Study_ID/Outcome_ID
##
## Test for Heterogeneity:
## Q(df = 126) = 682.6223, p-val < .0001
##
## Model Results:
##
## estimate      se    tval   df    pval    ci.lb   ci.ub
##   0.0494  0.0493  1.0021  126  0.3182  -0.0481  0.1469
##
## ---
## Signif. codes:  0 '***' 0.001 '**' 0.01 '*' 0.05 '.' 0.1 ' ' 1
```

```
     # Compare the two models
anova(threelevel.metafor, twolevel.metafor)
```

```
##
##         df     AIC     BIC    AICc   logLik    LRT   pval       QE
## Full     3 73.6962 82.2288 73.8914 -33.8481                682.6223
## Reduced  2 73.6432 79.3315 73.7399 -34.8216 1.9469 0.1629 682.6223
```

The smaller are AIC and BIC, the better the fit. Thus, the two-level has very slightly better fit indices than the three-level model. Yet, those differences are not significant (p=0.163)

Now, we are going to assess if there is an asymmetry and eventual publication bias existing in our dataset. However, as no function exists to directly do it with aggregated scores per study, we will pass by a univariate analysis deliberately enforcing simple means of effect sizes, SE, and variances.

```r
meta_means <- fullData %>% select(Study_ID, g, SE_g, V_g) %>%
  group_by(Study_ID) %>% summarise(mean_g = mean(g), mean_SE = mean(SE_g),
                                  mean_v = mean(V_g))

    # Add confidence intervals
meta_means$ci.lb <- meta_means$mean_g - 1.96 * sqrt(meta_means$mean_v)
meta_means$ci.ub <- meta_means$mean_g + 1.96 * sqrt(meta_means$mean_v)
meta_means
```

```
## # A tibble: 12 x 6
##    Study_ID   mean_g mean_SE mean_v   ci.lb   ci.ub
##       <int>    <dbl>   <dbl>  <dbl>   <dbl>   <dbl>
##  1        1  0.269     0.263 0.0692 -0.246   0.784
##  2        2  0.453     0.259 0.0673 -0.0557  0.961
##  3        3  0.288     0.132 0.0174  0.0294  0.547
##  4        4  0.224     0.170 0.0288 -0.109   0.557
##  5        5  0.169     0.172 0.0297 -0.169   0.507
##  6        6  0.142     0.239 0.0569 -0.325   0.610
##  7        7  0.117     0.222 0.0492 -0.318   0.552
##  8        8  0.128     0.201 0.0404 -0.266   0.522
##  9        9  0.0201    0.125 0.0156 -0.224   0.265
## 10       10  0.00625   0.209 0.0439 -0.404   0.417
## 11       11 -0.222     0.119 0.0141 -0.454   0.0105
## 12       12 -0.411     0.121 0.0147 -0.649  -0.174
```

```r
    # Univariate Meta-analysis from already computed effects size
m1 <-metagen(TE=g, seTE=SE_g, data=fullData, studlab=paste(Short_Reference), random = TRUE,
             method.tau = "HE", hakn = TRUE, prediction=TRUE, sm="SMD")
m1
```

```
## Number of studies: k = 127
##
##                               SMD            95%-CI  z|t  p-value
## Common effect model       0.0620 [ 0.0320; 0.0919] 4.06 < 0.0001
## Random effects model (HK) 0.1046 [ 0.0472; 0.1619] 3.61   0.0004
## Prediction interval               [-0.4366; 0.6458]
##
## Quantifying heterogeneity:
##  tau^2 = 0.0739 [0.0492; 0.1045]; tau = 0.2719 [0.2218; 0.3233]
##  I^2 = 70.2% [64.3%; 75.1%]; H = 1.83 [1.67; 2.00]
##
## Test of heterogeneity:
##       Q d.f.  p-value
##  422.73  126 < 0.0001
##
## Details on meta-analytical method:
## - Inverse variance method
## - Hedges estimator for tau^2
## - Q-Profile method for confidence interval of tau^2 and tau
## - Hartung-Knapp adjustment for random effects model (df = 126)
## - Prediction interval based on t-distribution (df = 125)
```

```r
    # double-check with metafor package
result.univariate.metafor<-rma(yi = g, sei=SE_g, data=fullData, slab = paste(Short_Reference),
                               method = "HE", test="knha")
```

```
result.univariate.metafor
```

```
##
## Random-Effects Model (k = 127; tau^2 estimator: HE)
##
## tau^2 (estimated amount of total heterogeneity): 0.0739 (SE = 0.0142)
## tau (square root of estimated tau^2 value):      0.2719
## I^2 (total heterogeneity / total variability):   71.33%
## H^2 (total variability / sampling variability):  3.49
##
## Test for Heterogeneity:
## Q(df = 126) = 422.7323, p-val < .0001
##
## Model Results:
##
## estimate      se    tval   df    pval   ci.lb   ci.ub
##   0.1046  0.0290  3.6077  126  0.0004  0.0472  0.1619  ***
##
## ---
## Signif. codes:  0 '***' 0.001 '**' 0.01 '*' 0.05 '.' 0.1 ' ' 1
```

```
#==> perfect, same result

    # Entry data is the simple means data
entry_data <- as.data.frame(meta_means[, c("mean_g","mean_SE")])

    # Run Egger's regression (separately) and extract estimates, etc.
eggers_regression <- metabias(x = entry_data$mean_g,
                              seTE = entry_data$mean_SE,
                              method.bias = "Egger",
                              plotit = F)
eggers_regression$estimate
```

```
##         bias      se.bias    intercept se.intercept
##    3.1335573    1.3972840   -0.4802917    0.2296435
```

```
eggers_p_one_tailed <- eggers_regression$p.value/2
print(eggers_p_one_tailed)
```

```
## [1] 0.02439403
```

The Egger's test confirmed our assumed asymmetry (intercept=-0.48, p=0.024).

Let's now print a funnel plot with a regression line and perform a trim-and-fill analysis to evaluate how many studies are missing.

```
eggers_intercept <- eggers_regression$estimate[1]

   # Precompute on which side to fill studies in funnel plot
if (eggers_regression$estimate[[1]]<0) {
  fill_side = "right"
} else {
  fill_side = "left"
}
```

```r
   # Change label title to Hedges' g
Hedges_g <- expression(paste("Effect Size (Hedges' ", italic("g"), ")"))

   # Call metaviz funnel plot visualization and adapt with ggplot
p_funnel <- viz_funnel(x = entry_data, contours = T, sig_contours = F,
                       egger = T,
                       trim_and_fill = T,
                       trim_and_fill_side = fill_side,
                       xlab = Hedges_g,
                       ylab = "Standard Error")

p_funnel + theme_bw() +
  theme(axis.text.x = element_text(colour="grey20",size=13,angle=0,hjust=.5,vjust=0,
                                   face="bold"),
        axis.text.y = element_text(colour="grey20",size=13,angle=0,hjust=.5,vjust=0,
                                   face="bold"),
        axis.title.x = element_text(colour="grey20",size=13,angle=0,hjust=.5,vjust=0,
                                    face="bold"),
        axis.title.y = element_text(colour="grey20",size=13,hjust=.5,vjust=0,
                                    face="bold"))+
  ggtitle(paste("Funnel plot 5-HTT methylation in depressive individuals"))
```

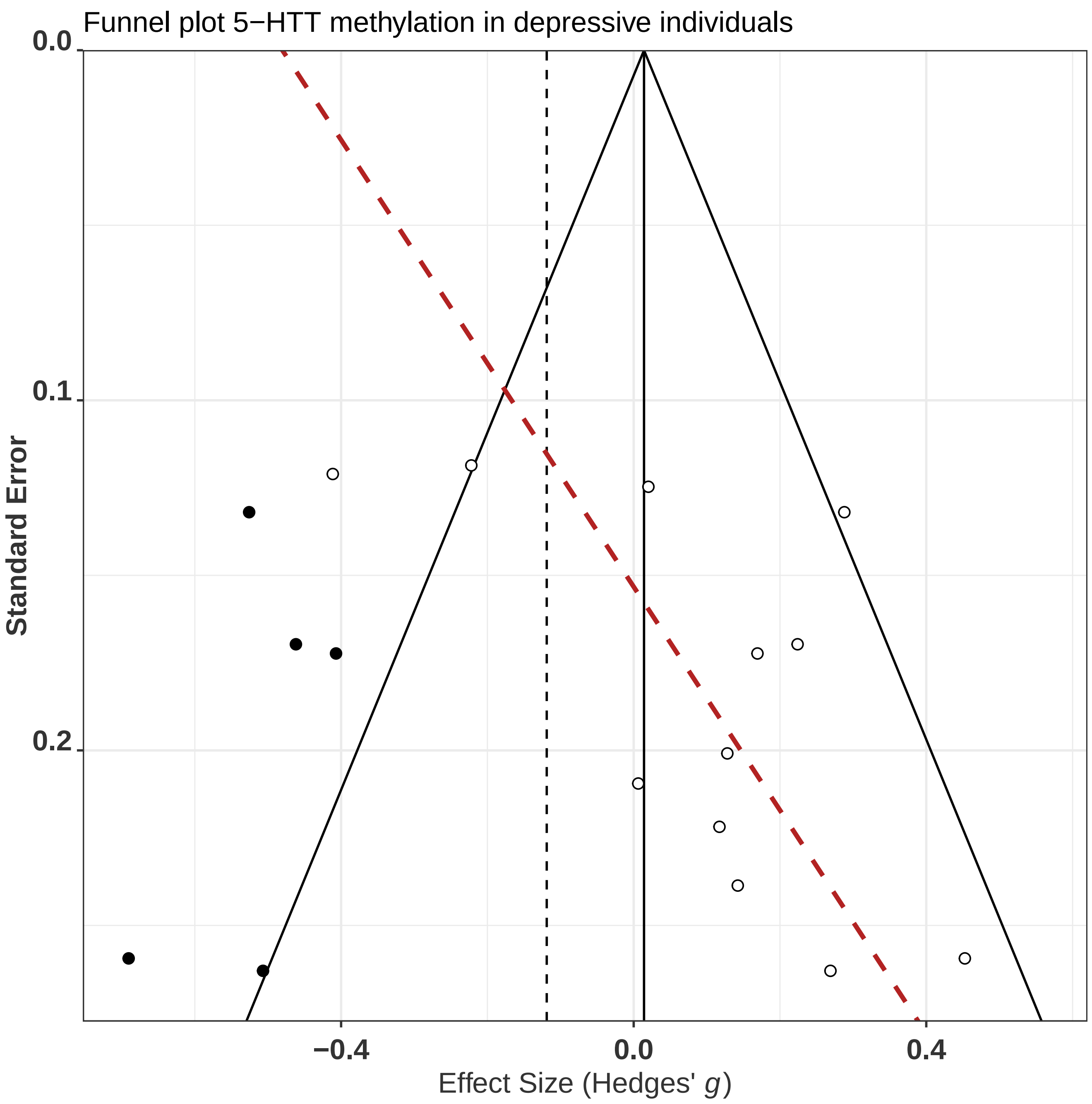


```
# Run trim and fill analysis separately and extract outcomes
trim_fill_data <- trimfill(x = entry_data$mean_g, seTE = entry_data$mean_SE,
                           method.tau ="REML",
                           ma.fixed = 1, # enforce fixed effect model
                           comb.fixed = 1,
                           comb.random = 1)



adjusted_g_random <- trim_fill_data$TE.random
adjusted_g_random_lower <- trim_fill_data$lower.random
adjusted_g_random_upper <- trim_fill_data$upper.random

print(adjusted_g_random)
```

```
## [1] -0.09135376
```

```r
print(adjusted_g_random_lower)
```

```
## [1] -0.2520463
```

```r
print(adjusted_g_random_upper)
```

```
## [1] 0.06933876
```

```r
nr_studies_filled <- trim_fill_data$k0
print(nr_studies_filled)
```

```
## [1] 5
```

The trim-and-fill analysis suggests five missing studies on the left side, providing a small negative adjusted Hedges' g (g=-0.09, 95% CI: -0.25 to 0.06).

2.2 Moderator analysis

Due to the minimal heterogeneity between studies, a between-studies moderator analysis was not conducted. However, as the within study was moderate (I2(2)=58%), an analysis was performed to assess the independent effects of individual CpG sites. To do so, only studies that examined single CpG sites were retained in the analysis (see paper). Besides, considering the large number of CpG sites that were analysed only once, only those evaluated at least 3 times were included in the analysis. This filtering reduced the dataset to 7 studies and 33 effect sizes.

Let's first recharge this new data and re-do the main analysis to verify that still some heterogeneity remains.

```r
     # To upload the file from local storage
fullData_bis<-read.csv("C:/Users/Florian/Downloads/5HTT_group_comparison_singleCpG_FINAL.csv",
                    sep=";", dec=".")

     # To be sure that our values are considered as numeric values
fullData_bis$g<- as.numeric(fullData_bis$g)
fullData_bis$V_g<- as.numeric(fullData_bis$V_g)
fullData_bis$SE_g<- as.numeric(fullData_bis$SE_g)
fullData_bis$Age<- as.numeric(fullData_bis$Age)
fullData_bis$Perc_fem<- as.numeric(fullData_bis$Perc_fem)

     # Some sanity checks
unique_studies  <- unique(fullData_bis$Study_ID)  # unique studies
nr_studies  <- length(unique_studies)
nr_studies
```

```
## [1] 7
```

```r
     # Add an outcome ID for later multilevel modeling
fullData_bis$Outcome_ID <- seq(from = 1, to = nrow(fullData_bis), by = 1)

     # Add n_effects (number of effects per study) for later visualisation purposes
fullData_bis<-fullData_bis %>% group_by(Study_ID)%>% mutate(n_effects=n())
```

```r
    # Re-do analysis the 3-level meta-analysis without reduced sample

fullData_bis <-escalc(measure = "GEN", yi = g, vi = V_g, data = fullData_bis, ci = ,)

V<-vcalc(vi, cluster=Study_ID, obs=Outcome_ID, data=fullData_bis, rho=0.6)

threelevel.metafor_bis<-rma.mv(g, V,
                          random = ~ 1 | Study_ID/Outcome_ID,
                          tdist = TRUE,
                          test= "t",
                          data = fullData_bis,
                          method = "ML",
                          level=95)
summary(threelevel.metafor_bis)
```

```
##
## Multivariate Meta-Analysis Model (k = 33; method: ML)
##
##   logLik  Deviance       AIC       BIC      AICc
##  -6.2530   80.0000   18.5061   22.9956   19.3337
##
## Variance Components:
##
##             estim    sqrt  nlvls  fixed               factor
## sigma^2.1  0.0563  0.2372      7     no             Study_ID
## sigma^2.2  0.0409  0.2022     33     no  Study_ID/Outcome_ID
##
## Test for Heterogeneity:
## Q(df = 32) = 130.8068, p-val < .0001
##
## Model Results:
##
## estimate      se    tval  df    pval    ci.lb   ci.ub
##   0.1236  0.1162  1.0639  32  0.2953  -0.1131  0.3604
##
## ---
## Signif. codes:  0 '***' 0.001 '**' 0.01 '*' 0.05 '.' 0.1 ' ' 1
```

```r
i2<-var.comp(threelevel.metafor)
print(i2)
```

```
## $results
##         % of total variance    I2
## Level 1            25.30783   ---
## Level 2            58.04699 58.05
## Level 3            16.64519 16.65
##
## $totalI2
## [1] 74.69217
##
## $plot
```

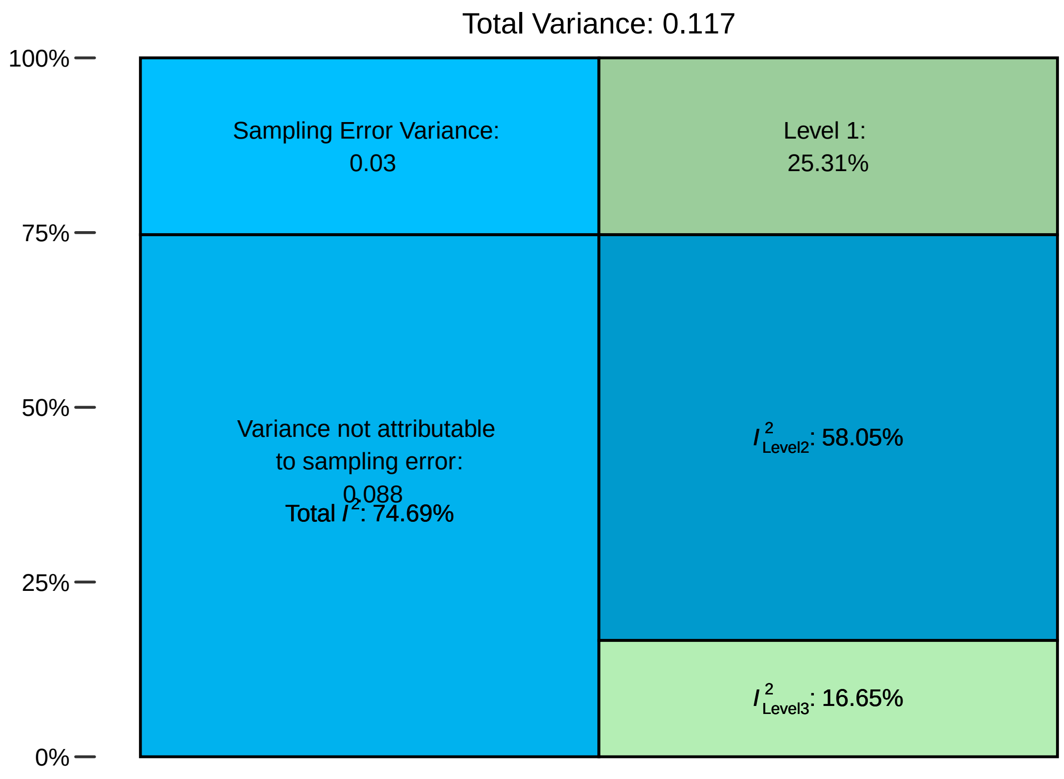


```
##
## attr(,"class")
## [1] "mlm.variance.distribution" "list"
```

The overall result is now slightly increased (g=0.12, 95% CI: -0.11 to 0.36) and the within-study heterogeneity decreased to 32.5% (for a total heterogeneity of 77.3%).

Let's now test if the single CpG analysis explains the left-over heterogeneity in our results.

```
threelevel.metafor_bis<-rma.mv(g, V,
                           mods= ~ CpG,
                           random = ~ 1 | Study_ID/Outcome_ID,
                           tdist = TRUE,
                           test= "t",
                           data = fullData_bis,
                           method = "ML",
                           level=95)
summary(threelevel.metafor_bis)
```

```
##
## Multivariate Meta-Analysis Model (k = 33; method: ML)
##
##   logLik  Deviance       AIC       BIC      AICc
##  -3.9483   75.3904   15.8966   21.8826   17.3251
##
## Variance Components:
##
##             estim    sqrt  nlvls  fixed              factor
```

```
## sigma^2.1  0.0596  0.2441      7     no             Study_ID
## sigma^2.2  0.0319  0.1786     33     no  Study_ID/Outcome_ID
##
## Test for Residual Heterogeneity:
## QE(df = 31) = 118.4148, p-val < .0001
##
## Test of Moderators (coefficient 2):
## F(df1 = 1, df2 = 31) = 5.0167, p-val = 0.0324
##
## Model Results:
##
##          estimate      se     tval  df    pval     ci.lb    ci.ub
## intrcpt  -13.7120  6.1762  -2.2201  31  0.0339  -26.3084  -1.1156  *
## CpG        0.0023  0.0010   2.2398  31  0.0324    0.0002   0.0044  *
##
## ---
## Signif. codes:  0 '***' 0.001 '**' 0.01 '*' 0.05 '.' 0.1 ' ' 1
```

While, the average effects between the tested CpG vary from -0.11 to 0.44, the results are not significant (QM=2.19, p=.070)

3. Depression severity and 5-HTT promoter methylation

3.1 Main analysis

3.1.1 Charge the dataset

Alright, let's now do the same thing for the second meta-analysis. Let's now charge the dataset from local storage. We are going to call it "fullData2" and ensure that all variables are properly considered (e.g., numeric, character). We are also going to proceed to some sanity checks such as checking the number of studies and outcomes (creating an outcome ID is also useful for later visualisation purposes). Finally, we are going to compute the number of outcomes per study and keep it in our dataset

```
   # Select data file to read
fullData2<-read.csv("C:/Users/Florian/Downloads/5HTT_correlation_nocorrection_FINAL.csv",
                    sep=";", dec=".")

   # To be sure that our values are considered as numeric values
fullData2$Z_r<- as.numeric(fullData2$Z_r)
fullData2$V_zr<- as.numeric(fullData2$V_zr)
fullData2$Se_zr<- as.numeric(fullData2$Se_zr)
fullData2$Age<- as.numeric(fullData2$Age)
fullData2$Perc_fem<- as.numeric(fullData2$perc_female)

   # Some sanity checks
unique_studies  <- unique(fullData2$Study_ID)  # unique studies
nr_studies  <- length(unique_studies)
nr_studies
```

```
## [1] 14
```

```
   # Add an outcome ID for later multilevel modeling
fullData2$Outcome_ID <- seq(from = 1, to = nrow(fullData2), by = 1)
```

```
   # Add n_effects (number of effects per study) for later visualisation purposes
fullData2<-fullData2 %>% group_by(Study_ID)%>% mutate(n_effects=n())
```

As for the previous meta-analysis, the first thing to do create an escalc object and construct the variance/covariance matrix. As we already saw how it works, I am not going to print it below (refer R script)

```
fullData2 <-escalc(measure = "GEN", yi = Z_r, vi = V_zr, data = fullData2, ci = ,)

V<-vcalc(vi, cluster=Study_ID, obs=Outcome_ID, data=fullData2, rho=0.6)
```

3.1.2 Three-level meta-analysis

Let's directly see the main analysis.

```
threelevel.metafor<-rma.mv(Z_r, V_zr,
                           random = ~ 1 | Study_ID/Outcome_ID,
                           tdist = TRUE,
                           test= "t",
                           data = fullData2,
                           method = "ML",
                           level=95)
summary(threelevel.metafor)
```

```
##
## Multivariate Meta-Analysis Model (k = 116; method: ML)
##
##    logLik   Deviance        AIC        BIC       AICc
##   78.3714   145.7165  -150.7429  -142.4821  -150.5286
##
## Variance Components:
##
##              estim    sqrt  nlvls  fixed               factor
## sigma^2.1  0.0159  0.1261     14     no             Study_ID
## sigma^2.2  0.0000  0.0000    116     no  Study_ID/Outcome_ID
##
## Test for Heterogeneity:
## Q(df = 115) = 184.4347, p-val < .0001
##
## Model Results:
##
## estimate      se    tval   df    pval    ci.lb   ci.ub
##   0.0457  0.0376  1.2162  115  0.2264  -0.0287  0.1201
##
## ---
## Signif. codes:  0 '***' 0.001 '**' 0.01 '*' 0.05 '.' 0.1 ' ' 1
```

Overall, 14 studies reporting 116 effect sizes are available for analysis. The average effect size for depression occurrence and 5-HTT promoter methylation across the 14 studies is null (Hedges' g = 0.05, 95% CI: -0.03 to 0.12, p=.226).

Let's now compute its prediction interval as index of the overall heterogeneity.

```
predict(threelevel.metafor, intervall="prediction")
```

```
##
##    pred     se   ci.lb  ci.ub   pi.lb  pi.ub
##  0.0457 0.0376 -0.0287 0.1201 -0.2150 0.3064
```

According to the prediction interval, in the universe of populations represented by the included studies, the true effect, in 95% of cases, will fall between small negative and small to moderate positive (g = -0.22 to 0.31).

Let's create a forest plot to visualise all the effects. We are going to organise it by the size of the effects and put a dotted line between each study to ease the reading.

```
dd <- c(0,diff(fullData2$Study_ID))
dd[dd < 0] <- 1
rows <- (1:116) + cumsum(dd)

par(tck=-0.01, mgp = c(1.6,0.2,0), mar=c(3,8,0,6))
forest(threelevel.metafor, cex=0.6, mlab= "RE Effect", header="First Author, Year, Effect",
       shade="zebra",rows = rows, cex.axis = 0.8, cex.lab = 0.8,xlab = "Z score",
       slab=paste(Short_Reference, year, " effect", esid, sep=","), ylim=c(0, max(rows)+3),
       efac=c(0,1),  xlim=c(-3,3), alim=c(-1, 1), at=seq(-1,3, by=1))
abline(h = rows[c(1,diff(rows)) == 2] - 1, lty="dotted")
```

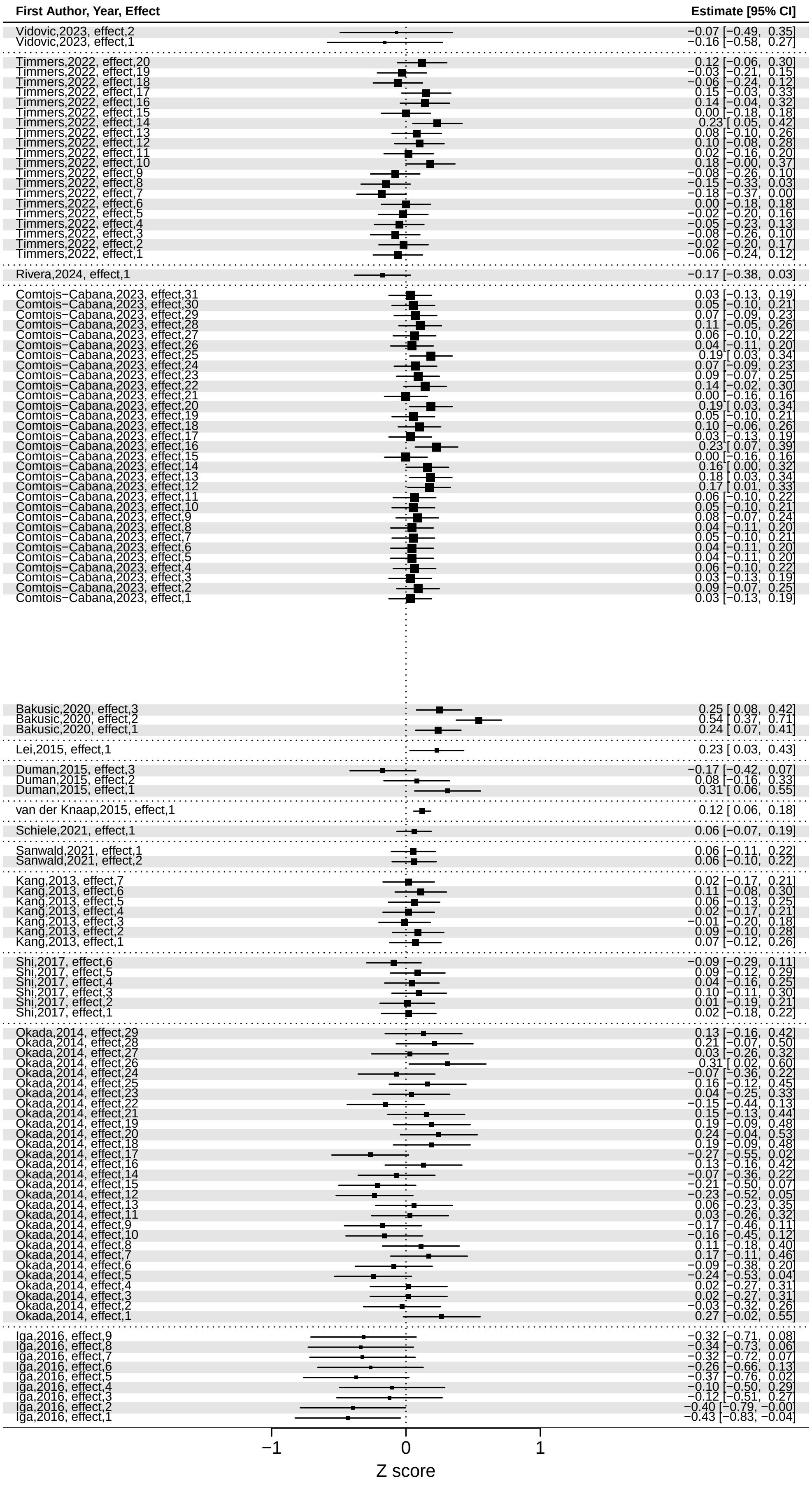
First Author, Year, Effect
Estimate [95% CI]
Vidovic,2023, effect,2 −0.07 [−0.49, 0.35]
Vidovic,2023, effect,1 −0.16 [−0.58, 0.27]
Timmers,2022, effect,20 0.12 [−0.06, 0.30]
Timmers,2022, effect,19 −0.03 [−0.21, 0.15]
Timmers,2022, effect,18 −0.06 [−0.24, 0.12]
Timmers,2022, effect,17 0.15 [−0.03, 0.33]
Timmers,2022, effect,16 0.14 [−0.04, 0.32]
Timmers,2022, effect,15 0.00 [−0.18, 0.18]
Timmers,2022, effect,14 0.23 [0.05, 0.42]
Timmers,2022, effect,13 0.08 [−0.10, 0.26]
Timmers,2022, effect,12 0.10 [−0.08, 0.28]
Timmers,2022, effect,11 0.02 [−0.16, 0.20]
Timmers,2022, effect,10 0.18 [−0.00, 0.37]
Timmers,2022, effect,9 −0.08 [−0.26, 0.10]
Timmers,2022, effect,8 −0.15 [−0.33, 0.03]
Timmers,2022, effect,7 −0.18 [−0.37, 0.00]
Timmers,2022, effect,6 0.00 [−0.18, 0.18]
Timmers,2022, effect,5 −0.02 [−0.20, 0.16]
Timmers,2022, effect,4 −0.05 [−0.23, 0.13]
Timmers,2022, effect,3 −0.08 [−0.26, 0.10]
Timmers,2022, effect,2 −0.02 [−0.20, 0.17]
Timmers,2022, effect,1 −0.06 [−0.24, 0.12]
Rivera,2024, effect,1 −0.17 [−0.38, 0.03]
Comtois−Cabana,2023, effect,31 0.03 [−0.13, 0.19]
Comtois−Cabana,2023, effect,30 0.05 [−0.10, 0.21]
Comtois−Cabana,2023, effect,29 0.07 [−0.09, 0.23]
Comtois−Cabana,2023, effect,28 0.11 [−0.05, 0.26]
Comtois−Cabana,2023, effect,27 0.06 [−0.10, 0.22]
Comtois−Cabana,2023, effect,26 0.04 [−0.11, 0.20]
Comtois−Cabana,2023, effect,25 0.19 [0.03, 0.34]
Comtois−Cabana,2023, effect,24 0.07 [−0.09, 0.23]
Comtois−Cabana,2023, effect,23 0.09 [−0.07, 0.25]
Comtois−Cabana,2023, effect,22 0.14 [−0.02, 0.30]
Comtois−Cabana,2023, effect,21 0.00 [−0.16, 0.16]
Comtois−Cabana,2023, effect,20 0.19 [0.03, 0.34]
Comtois−Cabana,2023, effect,19 0.05 [−0.10, 0.21]
Comtois−Cabana,2023, effect,18 0.10 [−0.06, 0.26]
Comtois−Cabana,2023, effect,17 0.03 [−0.13, 0.19]
Comtois−Cabana,2023, effect,16 0.23 [0.07, 0.39]
Comtois−Cabana,2023, effect,15 0.00 [−0.16, 0.16]
Comtois−Cabana,2023, effect,14 0.16 [0.00, 0.32]
Comtois−Cabana,2023, effect,13 0.18 [0.03, 0.34]
Comtois−Cabana,2023, effect,12 0.17 [0.01, 0.33]
Comtois−Cabana,2023, effect,11 0.06 [−0.10, 0.22]
Comtois−Cabana,2023, effect,10 0.05 [−0.10, 0.21]
Comtois−Cabana,2023, effect,9 0.08 [−0.07, 0.24]
Comtois−Cabana,2023, effect,8 0.04 [−0.11, 0.20]
Comtois−Cabana,2023, effect,7 0.05 [−0.10, 0.21]
Comtois−Cabana,2023, effect,6 0.04 [−0.11, 0.20]
Comtois−Cabana,2023, effect,5 0.04 [−0.11, 0.20]
Comtois−Cabana,2023, effect,4 0.06 [−0.10, 0.22]
Comtois−Cabana,2023, effect,3 0.03 [−0.13, 0.19]
Comtois−Cabana,2023, effect,2 0.09 [−0.07, 0.25]
Comtois−Cabana,2023, effect,1 0.03 [−0.13, 0.19]
Bakusic,2020, effect,3 0.25 [0.08, 0.42]
Bakusic,2020, effect,2 0.54 [0.37, 0.71]
Bakusic,2020, effect,1 0.24 [0.07, 0.41]
Lei,2015, effect,1 0.23 [0.03, 0.43]
Duman,2015, effect,3 −0.17 [−0.42, 0.07]
Duman,2015, effect,2 0.08 [−0.16, 0.33]
Duman,2015, effect,1 0.31 [0.06, 0.55]
van der Knaap,2015, effect,1 0.12 [0.06, 0.18]
Schiele,2021, effect,1 0.06 [−0.07, 0.19]
Sanwald,2021, effect,1 0.06 [−0.11, 0.22]
Sanwald,2021, effect,2 0.06 [−0.10, 0.22]
Kang,2013, effect,7 0.02 [−0.17, 0.21]
Kang,2013, effect,6 0.11 [−0.08, 0.30]
Kang,2013, effect,5 0.06 [−0.13, 0.25]
Kang,2013, effect,4 0.02 [−0.17, 0.21]
Kang,2013, effect,3 −0.01 [−0.20, 0.18]
Kang,2013, effect,2 0.09 [−0.10, 0.28]
Kang,2013, effect,1 0.07 [−0.12, 0.26]
Shi,2017, effect,6 −0.09 [−0.29, 0.11]
Shi,2017, effect,5 0.09 [−0.12, 0.29]
Shi,2017, effect,4 0.04 [−0.16, 0.25]
Shi,2017, effect,3 0.10 [−0.11, 0.30]
Shi,2017, effect,2 0.01 [−0.19, 0.21]
Shi,2017, effect,1 0.02 [−0.18, 0.22]
Okada,2014, effect,29 0.13 [−0.16, 0.42]
Okada,2014, effect,28 0.21 [−0.07, 0.50]
Okada,2014, effect,27 0.03 [−0.26, 0.32]
Okada,2014, effect,26 0.31 [0.02, 0.60]
Okada,2014, effect,24 −0.07 [−0.36, 0.22]
Okada,2014, effect,25 0.16 [−0.12, 0.45]
Okada,2014, effect,23 0.04 [−0.25, 0.33]
Okada,2014, effect,22 −0.15 [−0.44, 0.13]
Okada,2014, effect,21 0.15 [−0.13, 0.44]
Okada,2014, effect,19 0.19 [−0.09, 0.48]
Okada,2014, effect,20 0.24 [−0.04, 0.53]
Okada,2014, effect,18 0.19 [−0.09, 0.48]
Okada,2014, effect,17 −0.27 [−0.55, 0.02]
Okada,2014, effect,16 0.13 [−0.16, 0.42]
Okada,2014, effect,14 −0.07 [−0.36, 0.22]
Okada,2014, effect,15 −0.21 [−0.50, 0.07]
Okada,2014, effect,12 −0.23 [−0.52, 0.05]
Okada,2014, effect,13 0.06 [−0.23, 0.35]
Okada,2014, effect,11 0.03 [−0.26, 0.32]
Okada,2014, effect,9 −0.17 [−0.46, 0.11]
Okada,2014, effect,10 −0.16 [−0.45, 0.12]
Okada,2014, effect,8 0.11 [−0.18, 0.40]
Okada,2014, effect,7 0.17 [−0.11, 0.46]
Okada,2014, effect,6 −0.09 [−0.38, 0.20]
Okada,2014, effect,5 −0.24 [−0.53, 0.04]
Okada,2014, effect,4 0.02 [−0.27, 0.31]
Okada,2014, effect,3 0.02 [−0.27, 0.31]
Okada,2014, effect,2 −0.03 [−0.32, 0.26]
Okada,2014, effect,1 0.27 [−0.02, 0.55]
Iga,2016, effect,9 −0.32 [−0.71, 0.08]
Iga,2016, effect,8 −0.34 [−0.73, 0.06]
Iga,2016, effect,7 −0.32 [−0.72, 0.07]
Iga,2016, effect,6 −0.26 [−0.66, 0.13]
Iga,2016, effect,5 −0.37 [−0.76, 0.02]
Iga,2016, effect,4 −0.10 [−0.50, 0.29]
Iga,2016, effect,3 −0.12 [−0.51, 0.27]
Iga,2016, effect,2 −0.40 [−0.79, −0.00]
Iga,2016, effect,1 −0.43 [−0.83, −0.04]
−1
0
1
Z score

Nice. We can note that we still have the result of the multilevel modeling and that all the effects are not considered in a univariate manner. While it is not as obvious as for the previous meta-analysis, we can see the single CpG site effects can vary a lot.

Let's do the second forest with the aggregated scores per study.

```
agg_bis<-aggregate.escalc(fullData2, cluster=Study_ID, V=vcov(threelevel.metafor, type="obs"),
                         addk=TRUE)

res2<-rma(yi, vi, method="EE", data=agg_bis)

forest(res2, xlim=c(-4,5), mlab="RE Effect", slab=paste(Short_Reference, year, sep=", "),
       xlab = "Z score", shade="zebra", header=c("First Author, Year", "Pooled Estimates [95%]"),
       ilab=agg_bis$n_effects, ilab.xpos=-2, order = -res2$yi)
text(-2, res2$k+2, "n Estimates", font=2)
```

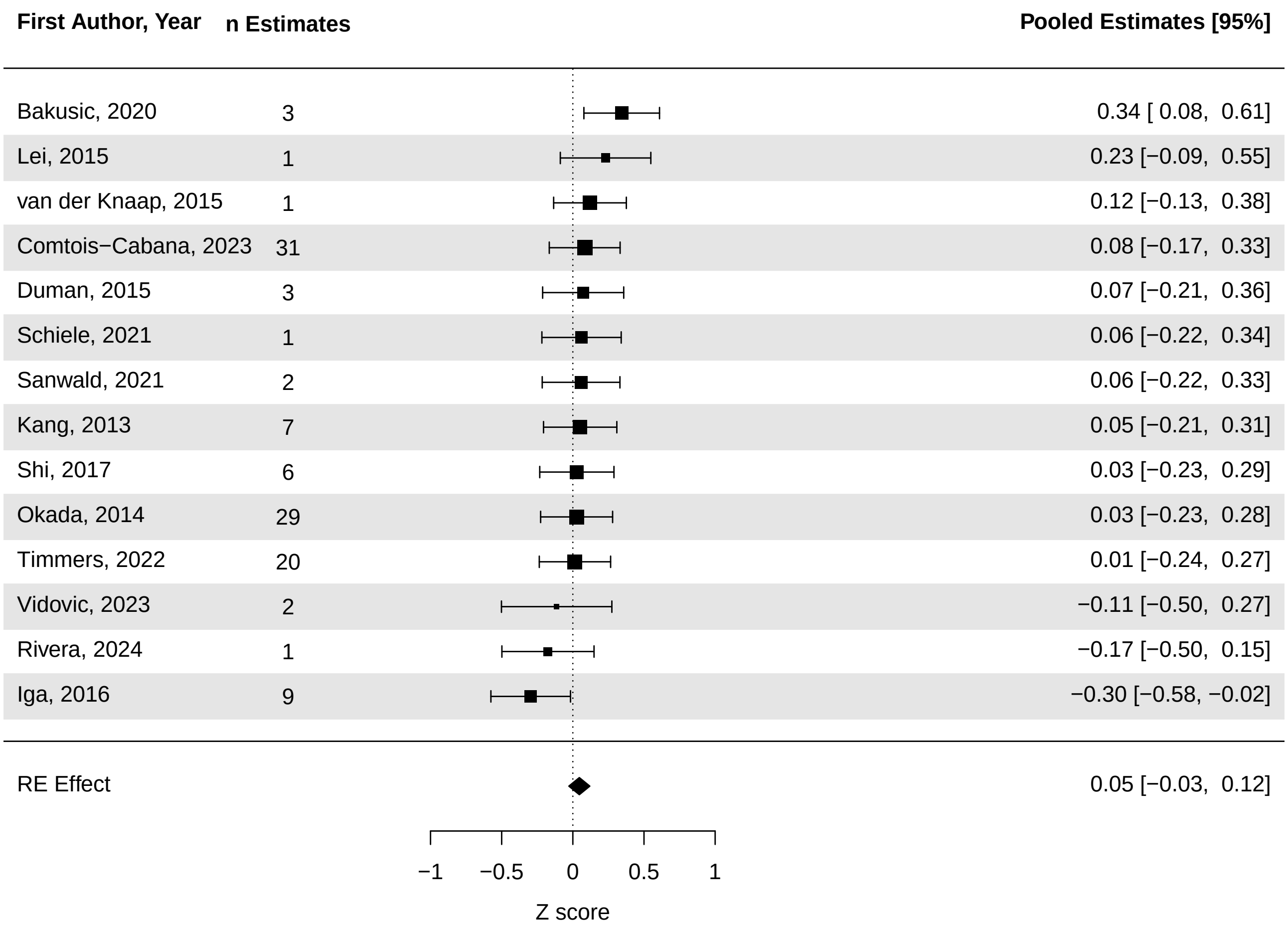

On this forest plot we can see that we have a bit more between-study heterogeneity than in the previous meta-analysis.

Let's evaluate the different levels of heterogeneity.

```
i2<-var.comp(threelevel.metafor)
print(i2)
```

```
## $results
```

```
##         % of total variance   I2
## Level 1        3.759697e+01  ---
## Level 2        6.454901e-08    0
## Level 3        6.240303e+01 62.4
##
## $totalI2
## [1] 62.40303
##
## $plot
```

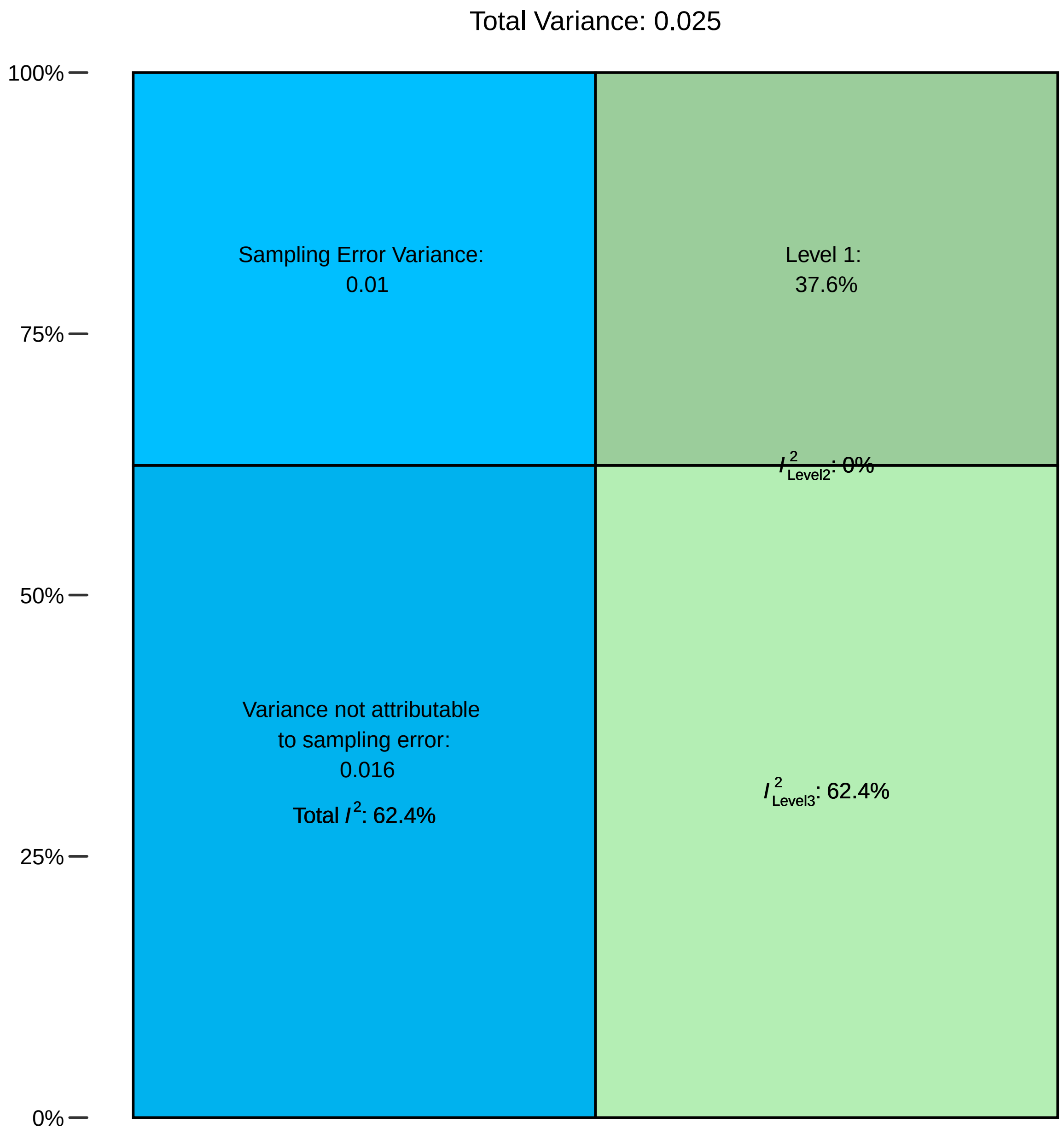


```
##
## attr(,"class")
## [1] "mlm.variance.distribution" "list"
```

The heterogeneity within-studies is null (T2(2)=0.000, I2(2)= 0%) and the heterogeneity between-studies (T2(3)=0.016, I2(3)= 62.4%) is moderate. Remark: The T2's were given when we did the main analysis.

Now, while the use of a three-level meta-analysis is justified from a data structure perspective, we can still compare it a two-level model to have a better idea of the fit of the chosen model.

```
twolevel.metafor<-rma.mv(Z_r, V_zr,
                         random = ~ 1 | Study_ID/Outcome_ID,
                         tdist = TRUE,
                         test= "t",
                         data = fullData2,
                         method = "ML",
                         sigma2=c(0, NA))

summary(twolevel.metafor)
```

```
##
## Multivariate Meta-Analysis Model (k = 116; method: ML)
##
##    logLik   Deviance        AIC        BIC       AICc
##   63.8398   174.7797  -123.6796  -118.1725  -123.5734
##
## Variance Components:
##
##              estim    sqrt  nlvls  fixed               factor
## sigma^2.1  0.0000  0.0000     14    yes             Study_ID
## sigma^2.2  0.0046  0.0679    116     no  Study_ID/Outcome_ID
##
## Test for Heterogeneity:
## Q(df = 115) = 184.4347, p-val < .0001
##
## Model Results:
##
## estimate      se    tval   df    pval   ci.lb   ci.ub
##   0.0526  0.0115  4.5608  115  <.0001  0.0298  0.0755  ***
##
## ---
## Signif. codes:  0 '***' 0.001 '**' 0.01 '*' 0.05 '.' 0.1 ' ' 1
```

```
   # Compare the two models
anova(threelevel.metafor, twolevel.metafor)
```

```
##
##         df       AIC       BIC      AICc  logLik     LRT   pval       QE
## Full     3 -150.7429 -142.4821 -150.5286 78.3714                184.4347
## Reduced  2 -123.6796 -118.1725 -123.5734 63.8398 29.0632 <.0001 184.4347
```

The three-level model has significantly better fit indices than the two-level model.

Now, we are going to assess if there is an asymmetry and eventual publication bias existing in our dataset. However, as no function exists to directly do it with aggregated scores per study, we will pass by a univariate analysis deliberately enforcing simple means of effect sizes, SE, and variances.

```
   # deliberately enforce simple means of effect sizes, SE, and variance
library(meta)
meta_means <- fullData2 %>% select(Study_ID, Z_r, V_zr, Se_zr) %>%
```

```
  group_by(Study_ID) %>% summarise(mean_z = mean(Z_r), mean_SE = mean(Se_zr),
                                   mean_v = mean(V_zr))

   # Add confidence intervals
meta_means$ci.lb <- meta_means$mean_z - 1.96 * sqrt(meta_means$mean_v)
meta_means$ci.ub <- meta_means$mean_z + 1.96 * sqrt(meta_means$mean_v)
meta_means
```

```
## # A tibble: 14 x 6
##    Study_ID  mean_z mean_SE  mean_v   ci.lb  ci.ub
##       <int>   <dbl>   <dbl>   <dbl>   <dbl>  <dbl>
##  1        0  0.343   0.0861 0.00741  0.175  0.512
##  2        1  0.230   0.102  0.0104   0.0299 0.430
##  3        2  0.0727  0.125  0.0156  -0.172  0.318
##  4        3  0.121   0.0327 0.00107  0.0565 0.185
##  5        4  0.0611  0.0655 0.00429 -0.0673 0.189
##  6        5  0.0579  0.0836 0.00699 -0.106  0.222
##  7        6  0.0516  0.0976 0.00952 -0.140  0.243
##  8        7  0.0283  0.104  0.0108  -0.175  0.232
##  9        8  0.0266  0.146  0.0213  -0.259  0.313
## 10        9 -0.296   0.2    0.04    -0.688  0.0959
## 11       10  0.0833  0.0808 0.00654 -0.0751 0.242
## 12       11 -0.175   0.107  0.0114  -0.384  0.0342
## 13       12  0.0149  0.0937 0.00877 -0.169  0.198
## 14       13 -0.115   0.216  0.0465  -0.537  0.308
```

```
fullData2<-fullData2 %>% group_by(Study_ID)%>% mutate(n_effects=n())
```

```
   # Univariate Meta-analysis from already computed effects size
m1 <-metagen(TE=Z_r, seTE=Se_zr, data=fullData2, studlab=paste(Short_Reference),
            random = TRUE, method.tau = "HE", hakn = TRUE, prediction=TRUE,
            sm="SMD")
m1
```

```
## Number of studies: k = 116
##
##                               SMD            95%-CI  z|t  p-value
## Common effect model       0.0616 [ 0.0439; 0.0794] 6.80 < 0.0001
## Random effects model (HK) 0.0474 [ 0.0219; 0.0729] 3.68   0.0004
## Prediction interval              [-0.1616; 0.2564]
##
## Quantifying heterogeneity:
##  tau^2 = 0.0109 [0.0029; 0.0142]; tau = 0.1046 [0.0534; 0.1193]
##  I^2 = 37.6% [21.7%; 50.3%]; H = 1.27 [1.13; 1.42]
##
## Test of heterogeneity:
##       Q d.f.  p-value
##  184.43  115 < 0.0001
##
## Details on meta-analytical method:
## - Inverse variance method
## - Hedges estimator for tau^2
## - Q-Profile method for confidence interval of tau^2 and tau
## - Hartung-Knapp adjustment for random effects model (df = 115)
## - Prediction interval based on t-distribution (df = 114)
```

```
   # double-check with metafor package
result.univariate.metafor<-rma(yi = Z_r, sei=Se_zr, data=fullData2,
                               slab = paste(Short_Reference), method = "HE", test="knha")

result.univariate.metafor
```

```
##
## Random-Effects Model (k = 116; tau^2 estimator: HE)
##
## tau^2 (estimated amount of total heterogeneity): 0.0109 (SE = 0.0036)
## tau (square root of estimated tau^2 value):      0.1046
## I^2 (total heterogeneity / total variability):   53.30%
## H^2 (total variability / sampling variability):  2.14
##
## Test for Heterogeneity:
## Q(df = 115) = 184.4347, p-val < .0001
##
## Model Results:
##
## estimate      se    tval   df    pval   ci.lb   ci.ub
##   0.0474  0.0129  3.6804  115  0.0004  0.0219  0.0729  ***
##
## ---
## Signif. codes:  0 '***' 0.001 '**' 0.01 '*' 0.05 '.' 0.1 ' ' 1
```

```
#==> perfect, same result
   # Entry data is the simple means data
entry_data <- as.data.frame(meta_means[, c("mean_z","mean_SE")])


   # Run Egger's regression (separately) and extract estimates, etc.
eggers_regression <- metabias(x = entry_data$mean_z,
                              seTE = entry_data$mean_SE,
                              method.bias = "Egger",
                              plotit = F)
eggers_regression$estimate
```

```
##         bias      se.bias    intercept se.intercept
##  -1.14892294   0.71686932   0.17139585   0.05650359
```

```
eggers_p_one_tailed <- eggers_regression$p.value/2
print(eggers_p_one_tailed)
```

```
## [1] 0.06749121
```

```
eggers_intercept <- eggers_regression$estimate[1]
```

The p value of the Egger's test is inferior to 0.1 (one-tailed test), thus asymmetry is detected.

Let's now print a funnel plot with a regression line and perform a trim-and-fill analysis to evaluate how many studies are missing.

```
eggers_intercept <- eggers_regression$estimate[1]
```

```r
# Precompute on which side to fill studies in funnel plot
if (eggers_regression$estimate[[1]]<0) {
  fill_side = "right"
} else {
  fill_side = "left"
}

# Change label title to Hedges' g
Hedges_g <- expression(paste("Effect Size (Hedges' ", italic("g"), ")"))

# Call metaviz funnel plot visualization and adapt with ggplot
p_funnel <- viz_funnel(x = entry_data, contours = T, sig_contours = F,
                       egger = T,
                       trim_and_fill = T,
                       trim_and_fill_side = fill_side,
                       xlab = Hedges_g,
                       ylab = "Standard Error")

p_funnel + theme_bw() +
  theme(axis.text.x = element_text(colour="grey20",size=13,angle=0,hjust=.5,vjust=0,
                                   face="bold"),
        axis.text.y = element_text(colour="grey20",size=13,angle=0,hjust=.5,vjust=0,
                                   face="bold"),
        axis.title.x = element_text(colour="grey20",size=13,angle=0,hjust=.5,vjust=0,
                                    face="bold"),
        axis.title.y = element_text(colour="grey20",size=13,hjust=.5,vjust=0,
                                    face="bold"))+
  ggtitle(paste("Funnel plot 5-HTT methylation in depressive individuals"))
```

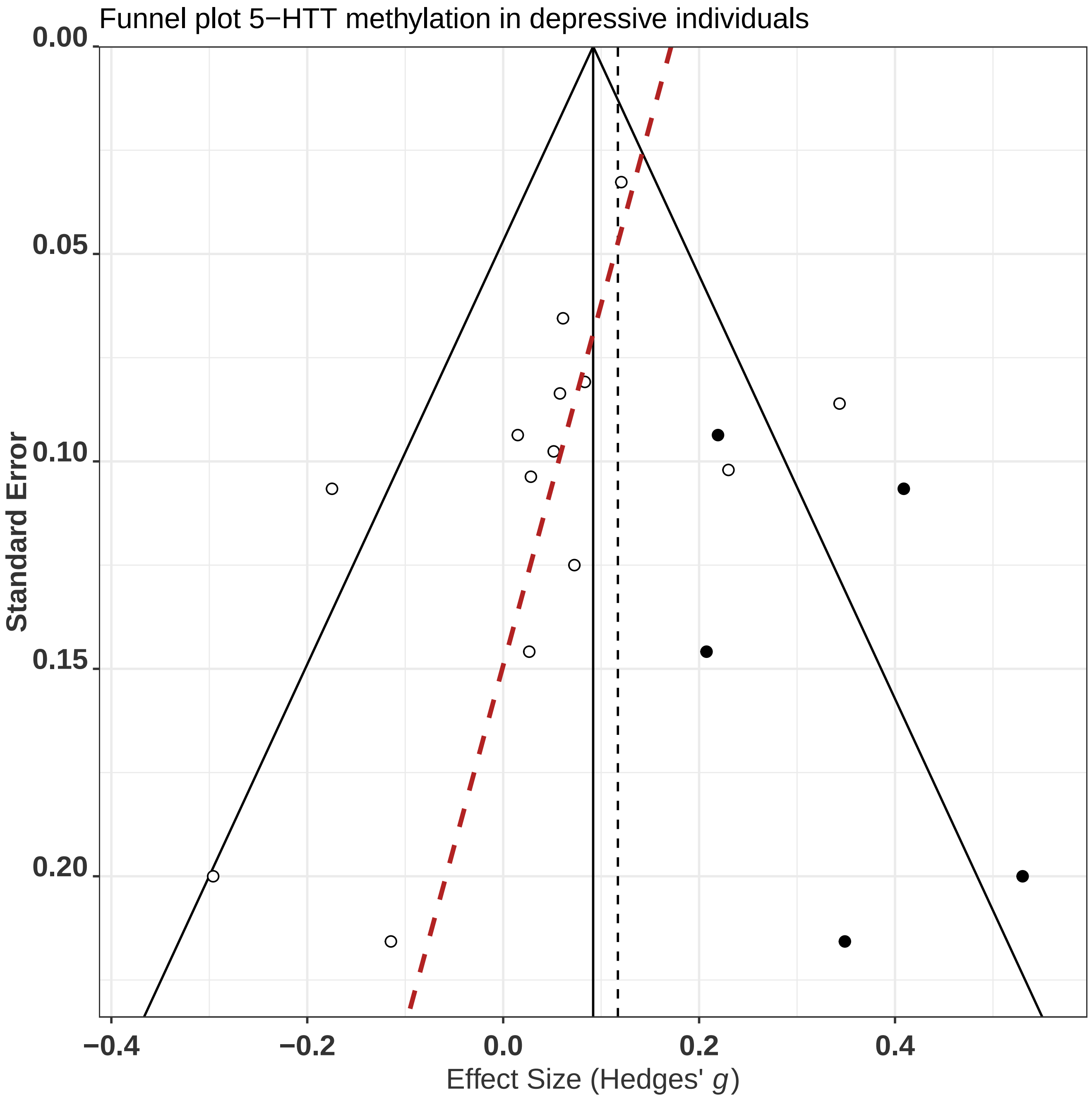


```r
# Run trim and fill analysis separately and extract outcomes
trim_fill_data <- trimfill(x = entry_data$mean_z, seTE = entry_data$mean_SE,
                           method.tau ="REML",
                           ma.fixed = 1, # enforce fixed effect model
                           comb.fixed = 1,
                           comb.random = 1)



adjusted_g_random <- trim_fill_data$TE.random
adjusted_g_random_lower <- trim_fill_data$lower.random
adjusted_g_random_upper <- trim_fill_data$upper.random

print(adjusted_g_random)
```

```
## [1] 0.117129
```

```
print(adjusted_g_random_lower)
```

```
## [1] 0.04627485
```

```
print(adjusted_g_random_upper)
```

```
## [1] 0.1879831
```

```
nr_studies_filled <- trim_fill_data$k0
print(nr_studies_filled)
```

```
## [1] 5
```

The trim-and-fill analysis suggests five missing studies not on the left side but on the right side, providing a slightly greater adjusted Hedges' g (g=0.12, 95% CI: 0.05 to 0.19).

3.2 Moderator analysis

There is a bit more than 62% of heterogeneity at study-level, thus we can do a moderator analysis.

3.2.1 Categorical moderators

Let's first check the categorical moderators, beginning with depression severity.

```
rma(yi, vi, method="EE", data=agg_bis, mods = ~ MDD_severity - 1)
```

```
##
## Fixed-Effects with Moderators Model (k = 14)
##
## I^2 (residual heterogeneity / unaccounted variability): 37.32%
## H^2 (unaccounted variability / sampling variability):   1.60
##
## Test for Residual Heterogeneity:
## QE(df = 9) = 14.3579, p-val = 0.1102
##
## Test of Moderators (coefficients 1:5):
## QM(df = 5) = 1.9740, p-val = 0.8527
##
## Model Results:
##
##                              estimate      se    zval    pval    ci.lb   ci.ub
## MDD_severityMild               0.0483  0.0639  0.7567  0.4492  -0.0768  0.1735
## MDD_severityModerate           0.0394  0.0672  0.5859  0.5579  -0.0923  0.1710
## MDD_severityModerate_Severe    0.0011  0.1139  0.0096  0.9924  -0.2222  0.2243
## MDD_severitySevere             0.0436  0.0971  0.4488  0.6536  -0.1468  0.2340
## MDD_severitySymptoms           0.1206  0.1303  0.9255  0.3547  -0.1348  0.3759
##
## MDD_severityMild
```

```
## MDD_severityModerate
## MDD_severityModerate_Severe
## MDD_severitySevere
## MDD_severitySymptoms
##
## ---
## Signif. codes:  0 '***' 0.001 '**' 0.01 '*' 0.05 '.' 0.1 ' ' 1
```

No group differences is detected between depressive symptoms but no diagnosed depression (k=1, g=0.12, 95% CI -0.13 to 0.38), mild (k=5, g=0.05, 95% CI -0.08 to 0.17), moderate (k=4, g=0.04, 95% CI -0.09 to 0.17), moderate to severe (k=2, g=0.01, 95% CI -0.22 to 0.22), and severe (k=2, g=0.04, 95% CI -0.17 to 0.23) depression (QM=1.97, df=4, p=.853). Yet, one can note that most of the categories are underrepresented.

What about the methylation techniques used?

```
rma(yi, vi, method="EE", data=agg_bis, mods = ~ methyl_type -1)
```

```
##
## Fixed-Effects with Moderators Model (k = 14)
##
## I^2 (residual heterogeneity / unaccounted variability): 31.84%
## H^2 (unaccounted variability / sampling variability):   1.47
##
## Test for Residual Heterogeneity:
## QE(df = 10) = 14.6722, p-val = 0.1445
##
## Test of Moderators (coefficients 1:4):
## QM(df = 4) = 1.6596, p-val = 0.7980
##
## Model Results:
##
##                          estimate      se    zval    pval    ci.lb   ci.ub
## methyl_typeBisulfite       0.0386  0.0854  0.4520  0.6513  -0.1288  0.2060
## methyl_typeMALDI           0.0694  0.0677  1.0249  0.3054  -0.0633  0.2022
## methyl_typeMicroarray      0.0312  0.1157  0.2694  0.7876  -0.1957  0.2580
## methyl_typePyro            0.0345  0.0599  0.5764  0.5643  -0.0828  0.1518
##
## ---
## Signif. codes:  0 '***' 0.001 '**' 0.01 '*' 0.05 '.' 0.1 ' ' 1
```

No group differences are detected between bisulfite sequencing (k=3, g=0.03, 95% CI -0.13 to 0.21), MALDI-TOF (k=4, g=0.07, 95% CI -0.06 to 0.20), microarray (k=2, g=0.03, 95% CI -0.20 to 0.26), and pyrosequencing techniques (k=5, g=0.03, 95% CI -0.08 to 0.15) (QM=1.66, df=3, p=.798).

Let's now check the type of sample used.

```
rma(yi, vi, method="EE", data=agg_bis, mods = ~ cell -1)
```

```
##
## Fixed-Effects with Moderators Model (k = 14)
##
## I^2 (residual heterogeneity / unaccounted variability): 7.99%
## H^2 (unaccounted variability / sampling variability):   1.09
##
## Test for Residual Heterogeneity:
## QE(df = 11) = 11.9547, p-val = 0.3671
```

```
##
## Test of Moderators (coefficients 1:3):
## QM(df = 3) = 4.3772, p-val = 0.2235
##
## Model Results:
##
##              estimate      se     zval    pval    ci.lb   ci.ub
## cellblood      0.0696  0.0426   1.6334  0.1024  -0.0139  0.1532
## cellSaliva     0.0833  0.1270   0.6564  0.5116  -0.1655  0.3322
## cellWBC       -0.1151  0.1018  -1.1306  0.2582  -0.3146  0.0844
##
## ---
## Signif. codes:  0 '***' 0.001 '**' 0.01 '*' 0.05 '.' 0.1 ' ' 1
```

No group differences in depression occurrence and 5-HTT promoter methylation are detected between peripheral blood (k=11, g=0.07, 95% CI -0.01 to 0.15), white blood cells (k=2, g=-0.12, 95% CI -0.31 to 0.08) and saliva (k=1, g=0.08, 95% CI -0.17 to 0.33) used for the methylation analysis (QM=4.38, df=2, p=.224).

We can also check if ethnicity moderate our results. Five studies were excluded from the analysis as they did not report this information. To maintain sufficient statistical power, ethnic backgrounds were reclassified into broader categories. Specifically, studies with individuals of Caucasian (k = 3) and Dutch (k = 1) descent were grouped under European ancestry; Chinese Han (k = 1) and Japanese (k = 2) under Asian ancestry; and African-American (k = 2) and Rwandan (k = 1) under African-American or African ancestry.

```
rma(yi, vi, method="EE", data=agg_bis, mods = ~ ethn -1)
```

```
##
## Fixed-Effects with Moderators Model (k = 14)
##
## I^2 (residual heterogeneity / unaccounted variability): 13.52%
## H^2 (unaccounted variability / sampling variability):   1.16
##
## Test for Residual Heterogeneity:
## QE(df = 10) = 11.5633, p-val = 0.3153
##
## Test of Moderators (coefficients 1:4):
## QM(df = 4) = 4.7686, p-val = 0.3119
##
## Model Results:
##
##              estimate      se     zval    pval    ci.lb   ci.ub
## ethn           0.1071  0.0590   1.8141  0.0697  -0.0086  0.2227  .
## ethnAsian     -0.0685  0.0777  -0.8818  0.3779  -0.2207  0.0838
## ethnEuro       0.0588  0.0743   0.7922  0.4283  -0.0867  0.2044
## ethnOthers     0.0312  0.1157   0.2694  0.7876  -0.1957  0.2580
##
## ---
## Signif. codes:  0 '***' 0.001 '**' 0.01 '*' 0.05 '.' 0.1 ' ' 1
```

No significant differences in depression occurrence or 5-HTT promoter methylation were observed between Asian ancestry (k = 3, Z = -0.07, 95% CI: -0.22 to 0.08), European ancestry (k = 4, Z = 0.0, 95% CI: -0.09 to 0.20), and African-American or African ancestry (k = 3, Z = 0.03, 95% CI: -0.20 to 0.26).

3.2.2 Continuous moderators

Let's now check the continuous moderators. Age first.

```r
rma(yi, vi, method="EE", data=agg_bis, mods = ~ Age)
```

```
##
## Fixed-Effects with Moderators Model (k = 14)
##
## I^2 (residual heterogeneity / unaccounted variability): 19.18%
## H^2 (unaccounted variability / sampling variability):   1.24
## R^2 (amount of heterogeneity accounted for):            0.00%
##
## Test for Residual Heterogeneity:
## QE(df = 12) = 14.8485, p-val = 0.2498
##
## Test of Moderators (coefficient 2):
## QM(df = 1) = 0.0041, p-val = 0.9492
##
## Model Results:
##
##          estimate      se     zval    pval    ci.lb   ci.ub
## intrcpt    0.0536  0.1295   0.4137  0.6791  -0.2003  0.3075
## Age       -0.0002  0.0033  -0.0637  0.9492  -0.0067  0.0063
##
## ---
## Signif. codes:  0 '***' 0.001 '**' 0.01 '*' 0.05 '.' 0.1 ' ' 1
```

The meta-regression reveals that the average sample age is not a significant moderator of the association between depression occurrence and 5-HTT promoter methylation (k=14, R2=0%, p=.949).

Let's finish with the percentage of female included in the tested sample.

```r
rma(yi, vi, method="EE", data=agg_bis, mods = ~ Perc_fem)
```

```
##
## Fixed-Effects with Moderators Model (k = 14)
##
## I^2 (residual heterogeneity / unaccounted variability): 19.01%
## H^2 (unaccounted variability / sampling variability):   1.23
## R^2 (amount of heterogeneity accounted for):            0.00%
##
## Test for Residual Heterogeneity:
## QE(df = 12) = 14.8167, p-val = 0.2516
##
## Test of Moderators (coefficient 2):
## QM(df = 1) = 0.0359, p-val = 0.8498
##
## Model Results:
##
##           estimate      se     zval    pval    ci.lb   ci.ub
## intrcpt     0.0604  0.0862   0.7004  0.4837  -0.1086  0.2293
## Perc_fem   -0.0003  0.0014  -0.1894  0.8498  -0.0030  0.0025
##
## ---
## Signif. codes:  0 '***' 0.001 '**' 0.01 '*' 0.05 '.' 0.1 ' ' 1
```

The meta-regression reveals that the female percentage in the tested sample is not a significant moderator of the association between depression occurrence and 5-HTT promoter methylation (k=14, R2=0%, p=.850).

4. Conclusion

The purpose of this report was to assist interested readers in navigating the analysis and the accompanying R script. Comprehensive details of the methods employed and the interpretation of results can be found in the main article.

Upon reviewing the analyses conducted in this report, it becomes evident that no clear association between 5-HTT promoter methylation and the occurrence or severity of depression were identified. In both cases, there existed heterogeneity in the effects observed within individual studies (one time not obvious because of overlap with sampling error), indicating diverse effects at specific CpG sites. Between-study heterogeneity was observable solely in the second meta-analysis, primarily driven by contradictory effects in two studies, though no clear moderator was distinctly highlighted
